# A Dynamic Simulation of Musculoskeletal Function in the Mouse Hindlimb During Trotting Locomotion

**DOI:** 10.3389/fbioe.2018.00061

**Published:** 2018-05-16

**Authors:** James P. Charles, Ornella Cappellari, John R. Hutchinson

**Affiliations:** ^1^Neuromuscular Diseases Group, Department of Comparative Biomedical Sciences, Royal Veterinary College, London, United Kingdom; ^2^Structure and Motion Lab, Department of Comparative Biomedical Sciences, Royal Veterinary College, Hatfield, United Kingdom

**Keywords:** rodent, biomechanics, muscle work, muscle function, kinematics

## Abstract

Mice are often used as animal models of various human neuromuscular diseases, and analysis of these models often requires detailed gait analysis. However, little is known of the dynamics of the mouse musculoskeletal system during locomotion. In this study, we used computer optimization procedures to create a simulation of trotting in a mouse, using a previously developed mouse hindlimb musculoskeletal model in conjunction with new experimental data, allowing muscle forces, activation patterns, and levels of mechanical work to be estimated. Analyzing musculotendon unit (MTU) mechanical work throughout the stride allowed a deeper understanding of their respective functions, with the rectus femoris MTU dominating the generation of positive and negative mechanical work during the swing and stance phases. This analysis also tested previous functional inferences of the mouse hindlimb made from anatomical data alone, such as the existence of a proximo-distal gradient of muscle function, thought to reflect adaptations for energy-efficient locomotion. The results do not strongly support the presence of this gradient within the mouse musculoskeletal system, particularly given relatively high negative net work output from the ankle plantarflexor MTUs, although more detailed simulations could test this further. This modeling analysis lays a foundation for future studies of the control of vertebrate movement through the development of neuromechanical simulations.

## Introduction

Many of the neuromuscular diseases that are studied using mice as a model organism, such as Duchenne Muscular Dystrophy (DMD) and Amyotrophic Lateral Sclerosis (ALS), are known to severely affect gait. The kinematics and kinetics of locomotion in mice have been the main area of interest in many studies investigating the progression of these diseases and the efficacy of potential treatments (Willmann et al., 2009; Henriques et al., 2010; Malerba et al., 2011; Sharp et al., 2011; Partridge, 2013; Brault et al., 2015). Furthermore, mice feature in many comparative biomechanics studies as a model organism for understanding the basic principles governing locomotion or muscle function/physiology, as a representative small organism for discovering general patterns of scaling of locomotor properties with body mass, and as an exemplar of a small mammal that might be close to the ancestral form, function and behavior of crown group mammals (O'Leary et al., 2013). Despite this prominence of mice in studies of locomotion, many details of muscle excitation patterns, muscle functions and fiber contractile dynamics during mouse locomotion remain unknown.

While detailed computerized biomechanical simulations have been used to estimate muscle forces and activations in human gait in many studies (Pandy, 2001; Zajac et al., 2003; Roberts and Belliveau, 2005; Liu et al., 2006; Lee and Piazza, 2009; McGowan et al., 2009; Miller et al., 2012; Steele et al., 2012; van der Krogt et al., 2012; Modenese et al., 2013; Pires et al., 2014), few similar studies into animal gait exist (Full and Ahn, 1995; Kargo et al., 2002; Merritt et al., 2008; Aoi et al., 2013; Sellers et al., 2013, 2017; Rankin et al., 2016). Factors such as joint moments, individual muscle forces, muscle contraction dynamics and muscle activation patterns during locomotion can be difficult or, depending on the subject, impossible to measure in a purely experimental context. The diversity and user-accessibility of such models and simulations mean that they have become, through refinements and validations, an increasingly reliable method with which to analyse the effects of various treatments for neuromuscular injuries or disorders, observe possible causes of various pathological gait patterns or simulate the effects of musculotendon surgical procedures, as well as to conduct basic scientific inquiries. Musculoskeletal modeling therefore is an opportunity to gain insight into muscle functions within the mouse musculoskeletal system, which could be used to inform new and/or improved animal models of neuromuscular diseases.

The functions of mouse hindlimb muscles have been described recently in a series of studies, by determining their architecture, geometry, and moment arms throughout specific joint rotations (Charles et al., 2016a,b). However, dynamic simulations and optimization techniques (Crowninshield and Brand, 1981; Modenese et al., 2013; Simpson et al., 2015) permit a deeper understanding of these functions, by estimating musculotendon (MTU) unit forces and activations, as well as the levels of mechanical work (force times length change; or energy) generated, transferred, or absorbed throughout dynamic movements (Dickinson et al., 2000; Biewener, 2011; Higham et al., 2011; Roberts et al., 2011; Syme and Shadwick, 2011; Rankin et al., 2016). Here we follow previous studies and general theory in maintaining a distinction between “muscle” (i.e., the living contractile unit or “belly” of striated/skeletal muscle tissue) and MTU (muscle plus tendinous and other passive tissue) structure and function, which is extremely important to maintain clarity about.

Static optimization is a commonly used computational technique for overcoming the problem of functional redundancy within vertebrate musculoskeletal systems; that is, multiple muscle excitation patterns are likely able to produce a certain movement (Crowninshield and Brand, 1981; Modenese et al., 2013; Simpson et al., 2015). This calculation estimates individual muscle activations and forces at each instant in time, typically with the goal of minimizing the sum of muscle activations squared but does not factor tendon energy transfer between time steps, or other historical factors such as delays between excitation and activation. However, several studies have shown that estimated muscle activity can be similar between static optimization and other dynamic simulation techniques (Anderson and Pandy, 2001; Lin et al., 2012; Morrow et al., 2014; Rankin et al., 2016), which typically do not have these assumptions.

These estimated forces and activations can be used to calculate MTU mechanical work, which can provide information regarding the flow of mechanical energy throughout the limb and is therefore useful for further discerning MTU functions. MTUs that generate high forces and positive power and mechanical work (concentric contraction) add energy to the system and can be classified as “motors.” “Brakes,” on the other hand, generate high forces but negative power and mechanical work (eccentric contraction), absorbing energy from the system. Some MTUs can generate high forces but little positive or negative mechanical work (i.e., remaining isometric), consequently acting as “struts” to stabilize joints. MTUs that act as “springs” generate high forces but switch between generating positive and negative work and power; producing close to zero net work. Similar biomechanical analyses and simulations have been used to investigate these individual MTU functions in animals employing a variety of locomotor modes (Biewener, 2011; Roberts et al., 2011; Syme and Shadwick, 2011; Rankin et al., 2016).

Here we describe the creation and analysis of a dynamic simulation of the pelvic appendage in a trotting mouse. With a previously built musculoskeletal model, we used static optimization and forward dynamics functions in a simulation that estimated MTU activation patterns and mechanical work output, with the aim to gain a deeper understanding of all major MTU functions in the mouse hindlimb during trotting locomotion.

## Materials and methods

### Limb kinematics and kinetics

**Figure 1:**
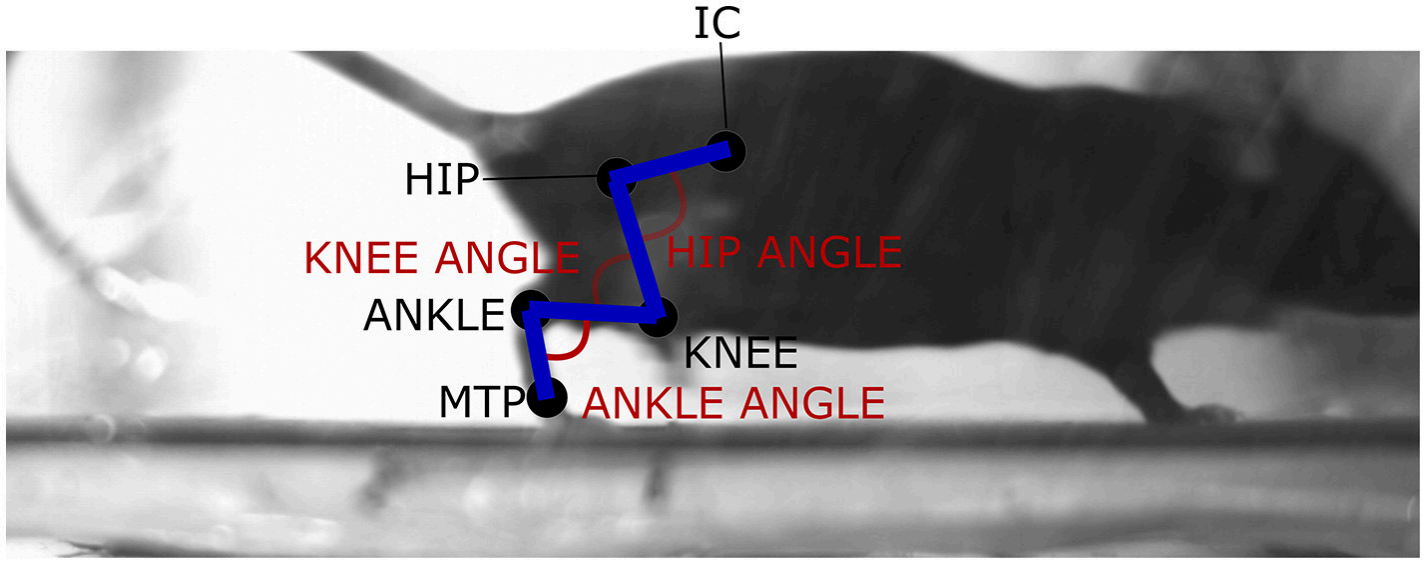
Sample video frame (right lateral view) of a mouse subject during trotting locomotion. The locations of the joint markers used to gather the hindlimb joint angle data for the hip, knee, ankle, and MTP joints, as well as pelvic tilt angle, are shown. IC, iliac crest; MTP, metatarsophalangeal joint.

The left and right hindlimbs of five C57BL/6 mice (male, mass: 18.7 ± 0.78 g, age: 42 days) were shaved, and the approximate centers of rotation of the hip, knee, ankle, and metatarsophalangeal (MTP) joints were estimated through palpation (based on the centers of rotation identified in Charles et al., 2016b) and marked on the skin with white enamel paint (Revell 14 ml White Gloss, RVL32104). An additional marker was also placed on the iliac crest, also known as the tuber coxae (Figure 1).

The mice were then placed on a custom-built trackway on top of a six-axis (3-force axes, 3-moment axes) custom-built strain gauge based acrylic force plate (7.5 × 7.5 cm, recording rate 2,500 Hz), and encouraged to trot to and from either end (average speed 0.59 ms^−1^). Each trial lasted 10 s and was filmed from both dorsal and lateral views (dorsal camera: GoPro Hero 4, 120 Hz; lateral camera: AOS S-PRI, 250 Hz imaging left/right sides depending on the direction of travel). Successful trials were those in which the near-side hindlimb completed a single stance phase with the entire foot in normal contact with a roughly 2 cm^2^ square hole in the center of the trackway, which allowed contact with the force plate (see Video S1 for a video of a trotting mouse). Trotting is the most frequently self-selected gait of mice (~0.5 ms^−1^) and so was analyzed here to ensure the biological relevance of the analysis (Smith et al., 2015).

The hindlimb kinematics for each successful trial were analyzed using a MATLAB (MathWorks, Natick, MA) routine developed by Hedrick (2008). The skin landmarks were digitized, obtaining their coordinates throughout each frame of a single stride within the trial. These raw coordinate data were filtered using a low-pass Butterworth filter (20 Hz). Using the digitized videos from the lateral camera, the values of pelvic tilt angle (the angle of the body segment relative to the horizontal axis of the ground segment), hip angle, knee angle, and angle ankle (in flexion-extension) throughout each frame of a single stride were calculated (metatarsophalangeal joint angles were not analyzed here and not incorporated into the model as a degree of freedom) using custom MATLAB scripts. Angles of hip adduction (i.e., the angle of the femur relative to the cranial-caudal midline plane) throughout single strides were calculated from digitized videos from the dorsal camera. Long-axis rotation (the third potential degree of freedom of each joint; ignoring translations) was presumed to be zero because this could not be reliably measured in the videos.

For each successful trial, cranial-caudal and dorsal-ventral ground reaction forces (GRFs) exerted by the hindlimb throughout the stance phase were calculated from the raw force plate data using a custom MATLAB software routine (including low-pass Butterworth filter, 20 Hz). Medio-lateral GRFs were collected but were judged too noisy to accurately include in our simulations. Filtering rate was deemed appropriate based on similar studies into small rodent GRFs (e.g., Zumwalt et al., 2006). The beginning and end of the stance phase were determined from the lateral camera's view and were defined as the frame in which the phalanges first contacted the force plate, and the frame in which they ceased contact with the force plate respectively. Due to inaccuracies of the force plate in determining the center of pressure (i.e., the point of application of the ground reaction force, CoP) of the small GRFs associated with the stance phase of mouse trotting, the CoP was assumed to be positioned at the mid-point of the distal end of the metatarsal bones, within the pedal (foot) segment (see Discussion for further interpretations of this assumption). All successful trials were analyzed, with steady-state locomotion assumed for all based on subjective evaluation of the videos.

**Figure 2:**
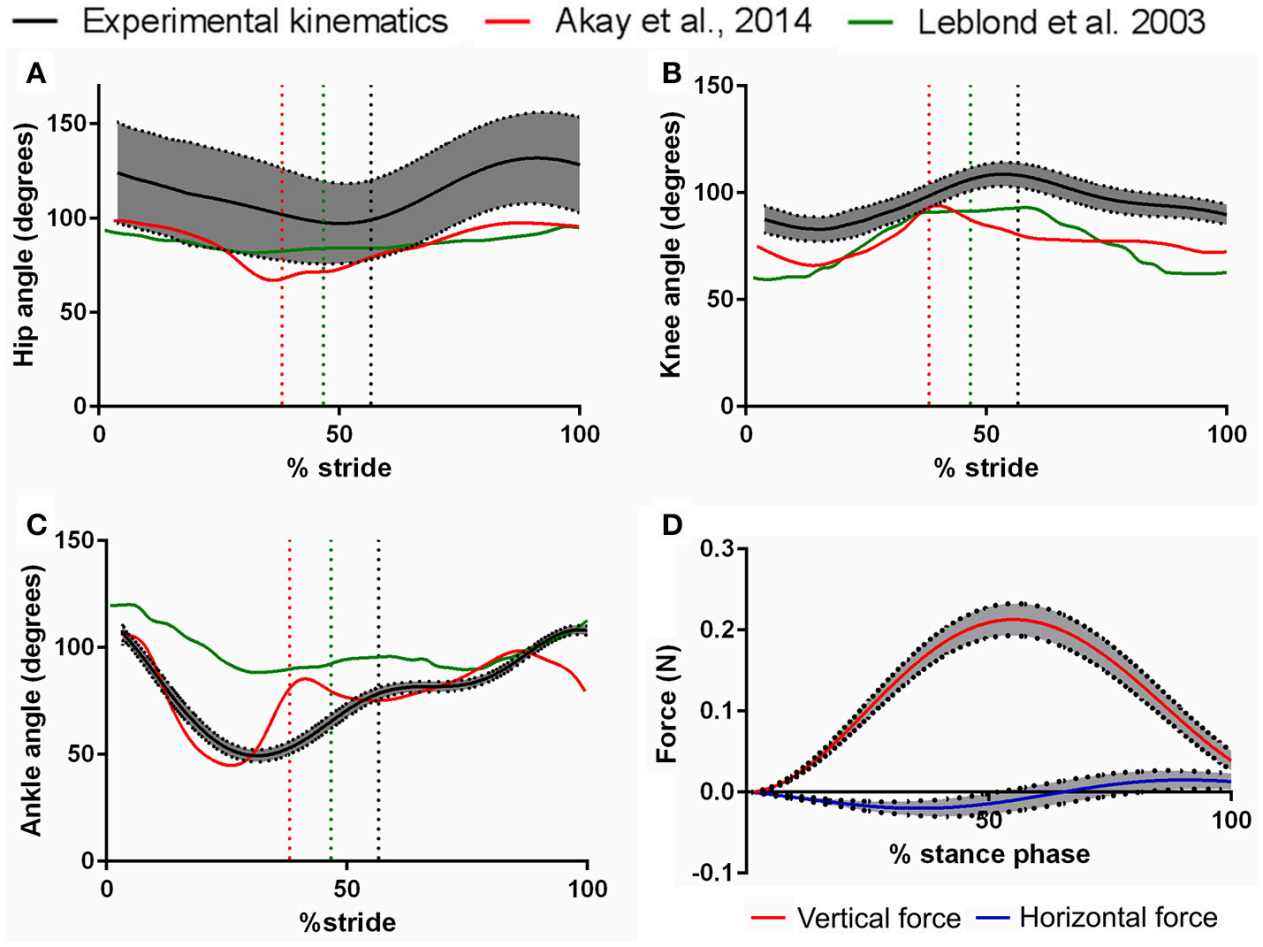
Mean angles (5 mice, 44 trials, ± 95% confidence intervals) of the hip **(A)**, knee **(B)**, and ankle **(C)** joints of the mouse hindlimb over one trotting stride, as measured with digitisation of markers from high-speed video. Walking hindlimb kinematics (mean traces) from Akay et al. (2014) and Leblond et al. (2003) are also shown for comparison and validation. Dashed vertical lines represent the swing-stance transitions of the corresponding stride (i.e., this study had longer swing phases from its faster speeds, relative to the other studies plotted here). Also shown in **(D)**: the mean (±95% confidence intervals) ground reaction forces exerted on the mouse hindlimb throughout the stance phase of one running stride, along the vertical (dorsal-ventral), and horizontal (cranial-caudal) axes.

Cubic spline interpolation was used to enable the calculation of mean values and 95% confidence intervals for the joint angles and GRF data from all of these trials (see Figure 2). Trotting speeds were estimated for all trials based on the distance traveled by the hip joint center's marker throughout the recorded gait cycles. The distribution of these trotting speeds was tested for normality using a Shapiro-Wilk Test, where a *P*-value > 0.05 indicates normal distribution.

### Creating a simulation

The musculoskeletal model of the mouse hindlimb and pelvis was created through a combination of diffusible iodine-based contrast-enhanced micro-CT (“diceCT”) scanning and microdissections (for full descriptions of the methods used to create the model, see Charles et al. (2016a) and Charles et al. (2016b)). Each MTU actuator in the model was based on a Hill-type muscle model (Zajac, 1989), where the force-length contractile dynamics of the muscle fibers and tendons were defined by muscle maximum isometric force, optimal fiber length, pennation angle, maximal contractile velocity and tendon slack length; as well as generic force-length and force-velocity properties (Millard et al., 2013) (see Table 1 for MTU properties).

**Table 1:** Force-generating parameters of the musculotendon units included in the hindlimb model.

**Functional group**	**Musculotendon unit**	**Abbreviation**	**F_max_ (N)**	**L_f_ (m)**	**L_ts_ (m)**	**Pennation angle (°)**
Hip rotators	Obturator externus	OE	0.086	0.00246	0.00096	0.00
	Obturator internus	OI	0.314	0.00565	0.00065	0.00
	Gemellus	GEM	0.179	0.00143	0.00001	0.00
	Quadratus femoris	QF	2.030	0.00465	0.00110	0.00
Hip adductors	Adductor magnus	AM	0.614	0.00760	0.00302	0.00
	Adductor longus	AL	0.402	0.00745	0.00255	0.00
	Adductor brevis	AB	0.234	0.00642	0.00176	0.00
	Gracilis posterior	GP	0.345	0.00912	0.00435	0.00
	Gracilis anterior	GA	0.402	0.00882	0.00607	0.00
Hip flexors	Psoas major	PMA	1.338	0.00697	0.00501	15.54
	Psoas minor	PMI	1.088	0.00578	0.00390	12.57
	Iliacus	ILI	0.549	0.00857	0.00275	0.00
	Pectineus	PECT	0.363	0.00277	0.00181	15.18
Hip extensors	Gluteus maximus (dorsal)	GM (d)	0.936	0.01305	0.00501	20.42
	Gluteus maximus (middle)	GM (m)	1.026	0.01271	0.00489	20.42
	Gluteus maximus (ventral)	GM (v)	1.049	0.01242	0.00478	20.42
	Caudofemoralis	CF	0.554	0.01137	0.00307	0.00
	Semimembranosus	SM	1.916	0.01165	0.00409	0.00
	Semitendinosus	ST	1.299	0.01111	0.00480	0.00
	Biceps femoris anterior	BFA	0.876	0.01145	0.00383	0.00
	Biceps femoris posterior (cranial)	BFP (cr)	0.725	0.01008	0.00491	0.00
	Biceps femoris posterior (middle)	BFP (m)	0.728	0.01004	0.00478	0.00
	Biceps femoris posterior (caudal)	BFP (ca)	0.611	0.01197	0.00406	0.00
Knee extensors	Rectus femoris	RF	4.162	0.00534	0.00853	15.89
	Vastus medialis	VM	1.098	0.00653	0.00768	16.15
	Vastus lateralis	VL	2.828	0.00681	0.00735	15.53
	Vastus intermedius	VI	0.367	0.00606	0.00702	10.92
Knee flexors	Popliteus	POP	0.307	0.00206	0.00203	0.00
Ankle dorsiflexors	Tibialis anterior	TA	2.422	0.00490	0.01180	16.58
	Extensor digitorum longus	EDL	0.368	0.00635	0.02378	12.39
	Extensor hallucis longus	EHL	0.069	0.00593	0.01793	9.56
Ankle plantarflexors	Medial gastrocnemius	MG	1.750	0.00550	0.01395	14.24
	Lateral gastrocnemius	LG	3.784	0.00541	0.01389	17.28
	Soleus	SOL	0.591	0.00316	0.00740	11.43
	Plantaris	PLANT	0.880	0.00431	0.01517	17.10
	Flexor digitorum longus	FDL	1.896	0.00431	0.02761	15.20
	Tibialis posterior	TP	0.549	0.00359	0.01500	15.44
Ankle everters	Peroneus longus	PL	0.647	0.00378	0.01408	14.90
	Peroneus tertius	PT	0.457	0.00339	0.01122	12.46
	Peroneus brevis	PB	0.396	0.00229	0.01005	11.46
	Peroneus digiti quarti	PDQA	0.112	0.00393	0.02357	12.42
	Peroneus digiti quinti	PDQI	0.102	0.00362	0.01959	9.44

*Maximum force (F_max_), fiber length (L_f_), and pennation angle (°) were derived from previously measured skeletal muscle architecture (Charles et al., 2016a). Tendon slack length (L_ts_) was estimated using a numerical optimization procedure from Manal and Buchanan (2004). Grouping was based on presumed functions during locomotion (Charles et al., 2016b)*.

**Figure 3:**
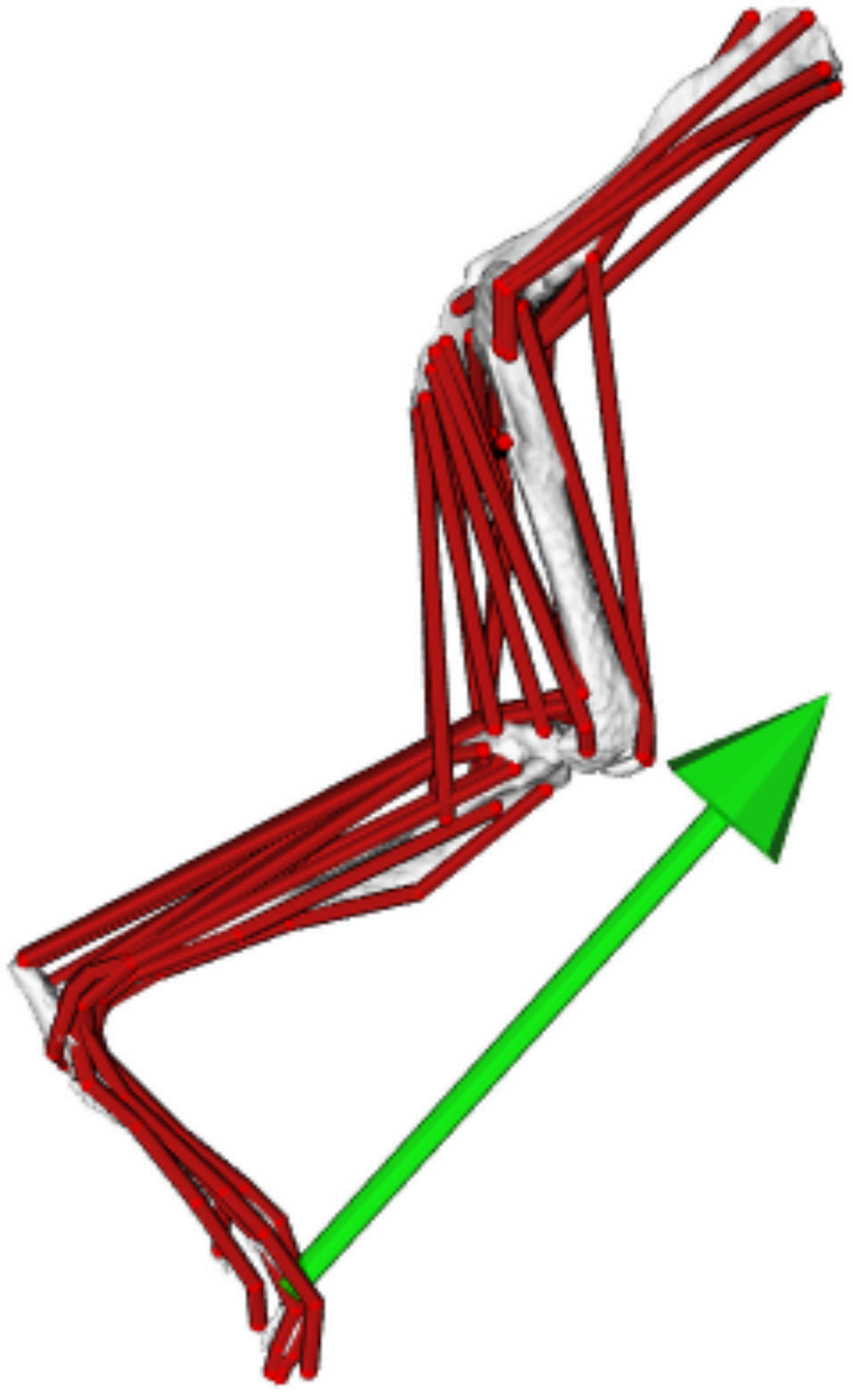
The musculoskeletal model of the mouse hindlimb and pelvis (right limb in lateral view), used here to simulate trotting locomotion. The green arrow represents the ground reaction force vector. For more details of the construction and validation of the model, see (Charles et al., 2016b).

To simulate mouse locomotion using this model (Figure 3), data from one representative trial, which fit within the 95% confidence intervals of the pooled mean values of both the joint angle and GRF data, were imported into open source biomechanical simulation software OpenSim (Delp et al., 2007). GRF data from the representative trial were normalized to the length of the whole stride, matching the kinematic data. The joint angles of the representative trial were converted to generalized coordinate values (gencoords) for use in the model, which expressed each joint angle relative to the fully extended reference pose of the model (see Charles et al., 2016b for more details). The kinematic and kinetic data used to create the simulation of trotting are available at https://simtk.org/home/mousehindlimb.

**Figure 4:**
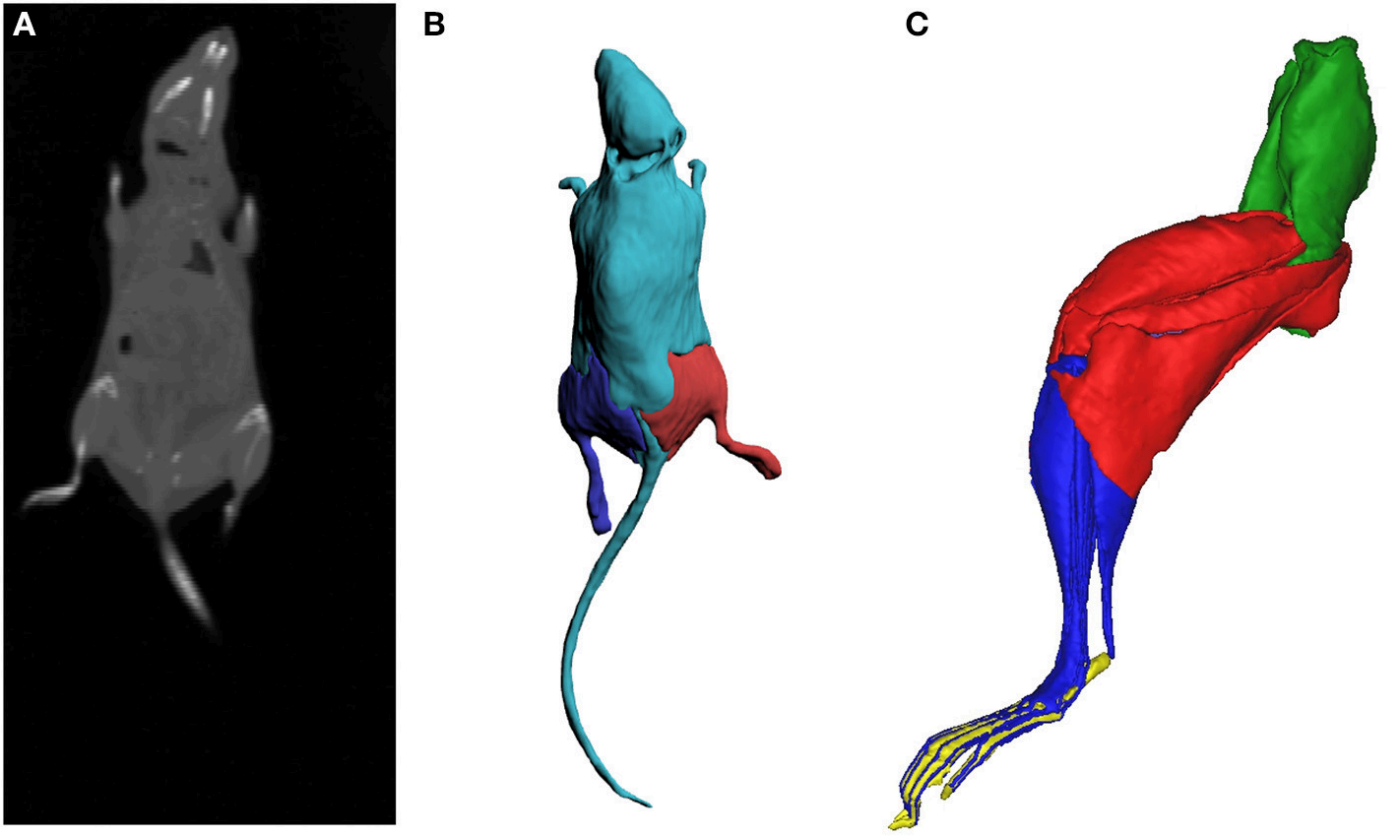
Full body CT scan slice of the mouse **(A)**, a stack of which was subsequently digitally segmented to create three-dimensional meshes of the body and the hindlimbs **(B)**, to estimate the mass and inertial properties of the body segments. The inertial properties of the hindlimb segments (**C**; oblique medial view) were calculated from digital segmentation of microCT images of the right hindlimb (see Charles et al., 2016a). Green, pelvis segment; Red, thigh segment; Blue, leg segment; Yellow, pedal segment.

To simulate the dynamics of locomotion, the mass and inertial properties of the different body segments of the mouse were obtained. A C57BL/6 mouse (mass: 24 g; age: 153 days) was euthanised by cervical dislocation and CT scanned (GE Medical Systems, Lightspeed Pro 16; 40 mA, 80 kV, voxel size: 0.188 mm) in a prone position with the body as straight as possible. The resulting reconstructed images were digitally segmented in Mimics software (Materialise Inc., Leuven, Belgium), where a three-dimensional (3D) mesh of the whole mouse body (minus the pelvis and right hindlimb) was created. The mass, center of mass (CoM) and inertial tensor around the CoM for each of the body segments were estimated from the 3D meshes using a custom MATLAB routine from Allen et al. (2013) and added to the musculoskeletal model (see Table 2 for values). Similar methods were used to estimate the properties of the thigh, leg, and foot segments of the right hindlimb. The CT scanning and digital segmentation of these hindlimb segments are described in detail in Charles et al. (2016b). The reconstructed CT scan data for the entire body and the 3D reconstruction of the body and right pelvic appendage are in Figure 4.

**Table 2:** The masses and inertial properties of the mouse torso and hindlimb segments used to simulate trotting in the hindlimb.

**Segment**	**Mass (g)**	**Center of mass (mm; x, y, z)**	**Moments of inertia (kgm^2^; xx, yy, zz)**
Torso	19.5	29.4	1.07 × 10^−6^
		−4.1	9.43 × 10^−6^
		−1.5	9.12 × 10^−6^
Pelvis	0.38	2.9	2.35 × 10^−9^
		−1.2	6.43 × 10^−9^
		2.4	6.70 × 10^−9^
Thigh	0.44	−2.8	6.83 × 10^−9^
		−7.2	4.23 × 10^−9^
		0.20	9.10 × 10^−9^
Lower Leg	0.21	−0.8	3.73 × 10^−9^
		−7.2	6.80 × 10^−10^
		0.0	4.10 × 10^−9^
Foot	0.063	4.5	6.10 × 10^−11^
		−0.20	1.26 × 10^−9^
		0.10	1.26 × 10^−9^

*Center of mass values are shown with respect to the coordinate system origins (i.e., proximally) of each segment (see Charles et al., 2016b for more details). + x, cranial; + y, proximal (dorsal); + z, lateral. See Figure 4 for how the segments were partitioned*.

Using the scaling tool within OpenSim, the muscle force-length and segment properties estimated above were scaled to match those of the experimental subject from which the limb kinematics and kinetics were derived. This scaling aimed to improve the consistency between experimentally derived joint angle and GRF data and the musculotendon dynamics of the musculoskeletal model. The segments of the model were scaled by the following factors (determined from the relative positions of each joint center marker from a lateral high-speed video frame): torso 0.936, pelvis 0.936, thigh 1.065, lower leg 0.926, and pedal 1.244.

Moment arms for each MTU (following the “virtual work” method as per Delp et al., 2007) throughout the simulated trotting stride were also calculated to indicate the effectiveness with which each MTU could carry out their presumed primary function (Charles et al., 2016a,b).

Inverse dynamics analysis, performed within OpenSim, estimated the net (extensor + flexor or abductor + adductor) joint moments at each joint, at each time point, that could produce the accelerations estimated by the measured joint angle and GRF data.

For this simulation, the knee and ankle joints were restricted to flexion-extension rotations, while the hip joint was restricted to both flexion-extension and adduction-abduction rotations. To assess the validity of our assumption of a fixed CoP for the application of the GRFs, the sensitivity of these joint moments, and by extension subsequent modeling estimates, to changes in CoP was tested by moving the CoP ± 2 mm along the cranial-caudal axis of the pedal segment. OpenSim's static optimization tool was used to resolve these net moments into individual maximal muscle moments and activations (1.0 = full activation, < 0.01 = no activation) that matched the experimental trotting data. The objective of the static optimization was to minimize the summed muscle activations squared at each time step. Each time step was solved independently, meaning that no energy was transferred between them (i.e., tendon energy storage and return), representing a purely static simplification (van der Krogt et al., 2012; Modenese et al., 2013; Rankin et al., 2016). Furthermore, passive fiber force generation was ignored, and tendons were assumed to be rigid, with all musculotendon unit changes occurring within the muscle fibers; a necessary assumption of the static optimization algorithm (see Video S2 for video of the static optimization simulation).

When performing the static optimization, residual and reserve actuators were appended to the model to account for any potential inconsistencies between the subject used to create the model and that used to gather the experimental data, or for any modeling assumptions (such as lack of trunk or forelimbs- see Discussion). This is a standard practice in simulations (Steele et al., 2012; van der Krogt et al., 2012; Modenese et al., 2013; Hicks et al., 2015; Rankin et al., 2016), and the actuators were only used to provide extra torque if the MTUs were unable to generate sufficient accelerations to satisfy the experimental data. Residual actuators, F_X_ and F_Y_, were assigned to the first free joint of the model (ground-to-body) and applied at the model's center of mass (CoM), whereas reserve actuators were added to each unlocked degree of freedom (pelvic tilt, hip flexion, hip adduction, knee extension, and ankle flexion). See Results for further information.

The activation of these reserve actuators within the static optimization routine incurred a high cost function within the simulation and were therefore only recruited if necessary. This ensured that the majority of each joint moment was produced by the muscle actuators. This cost was not applied to the residual actuators, which along with the pelvic tilt actuator functioned mainly to account for the lack of the diagonal forelimb in our model.

### MTU mechanical work

To estimate individual MTU mechanical work during trotting locomotion, the activations generated from the static optimization routine were used as direct inputs to OpenSim's muscle analysis function, which allows detailed MTU dynamics to be estimated from previously developed simulations. Here, the MTUs were constrained to their input activation levels and tendon compliance was accounted for. Positive and negative mechanical work for each MTU, as well as the reserve actuators, throughout the swing and stance phases and net work throughout the entire stride were calculated from this analysis by calculating the area under the actuator power curves, as in Rankin et al. (2016).

These estimates of net MTU work were compared to those calculated using the same method from actuator power curves generated by a forward dynamic simulation, an established method of estimating mechanical work (Neptune et al., 2004, 2009; Sasaki and Neptune, 2006). Here, muscle excitation-activation dynamics were modeled by first-order equations (Thelen et al., 2003), with activation and deactivation time constants of 10 and 40 ms respectively. Both excitation and activation levels were allowed to vary continuously between 0 (no excitation/activation) and 1 (full excitation/activation) throughout the simulation. Other muscle contractile dynamics (i.e., force generation, fiber lengths), could vary from those calculated in the static optimization.

The forward dynamics tool in OpenSim does not constrain the simulation to the experimental kinematics, unlike static optimization (Delp et al., 2007) and furthermore, does not force joint angles to be constrained “boundaries” during simulations. Instead, coordinate limit forces were added to each degree of freedom within the model (pelvic tilt, hip flexion, hip adduction, hip rotation, knee extension, ankle flexion, ankle adduction, and ankle inversion). These functioned to provide passive forces, of a defined stiffness, to their respective degree of freedom when the joint angles exceeded a defined upper or lower limit. The properties of these coordinate limit forces were chosen to restrict the joint angles to these limits (Table S1) and ensure that the joint angles of the forward dynamic simulations were as congruent as possible with those measured experimentally. The same residual and reserve actuators from the static optimization simulation were applied to the forward dynamic simulation.

## Results

### Experimental kinematics and kinetics

A total of 44 successful experimental trials were obtained from the five mice used to gather hindlimb kinematic and kinetic data. The average trotting speed was 0.59 ms^−1^ (duty factor: 0.44) over the analyzed trials, and these speeds were normally distributed (Shapiro-Wilk Test *P* value = 0.382). The pooled angular excursions of the hip, knee and ankle joints of the hindlimb throughout a single stride, and a comparison to previously published mouse hindlimb walking kinematic data, are shown in Figure 2.

Vertical GRFs (Figure 2D) on average peaked at 0.21 N (120% of body mass) at 55.1% of stance. In early stance phase, mean cranial-caudal force peaked at −0.02 N (10.9% of body mass), representing a braking (caudal) force at initial contact with the force plate. Later during stance, mean cranial-caudal force peaked at 0.015 N (8.19% of body mass), representing a propulsive (cranial) force as the foot pushed off the force plate. Mediolateral GRFs were too noisy to reliably quantify.

### Creating a simulation

**Figure 5:**
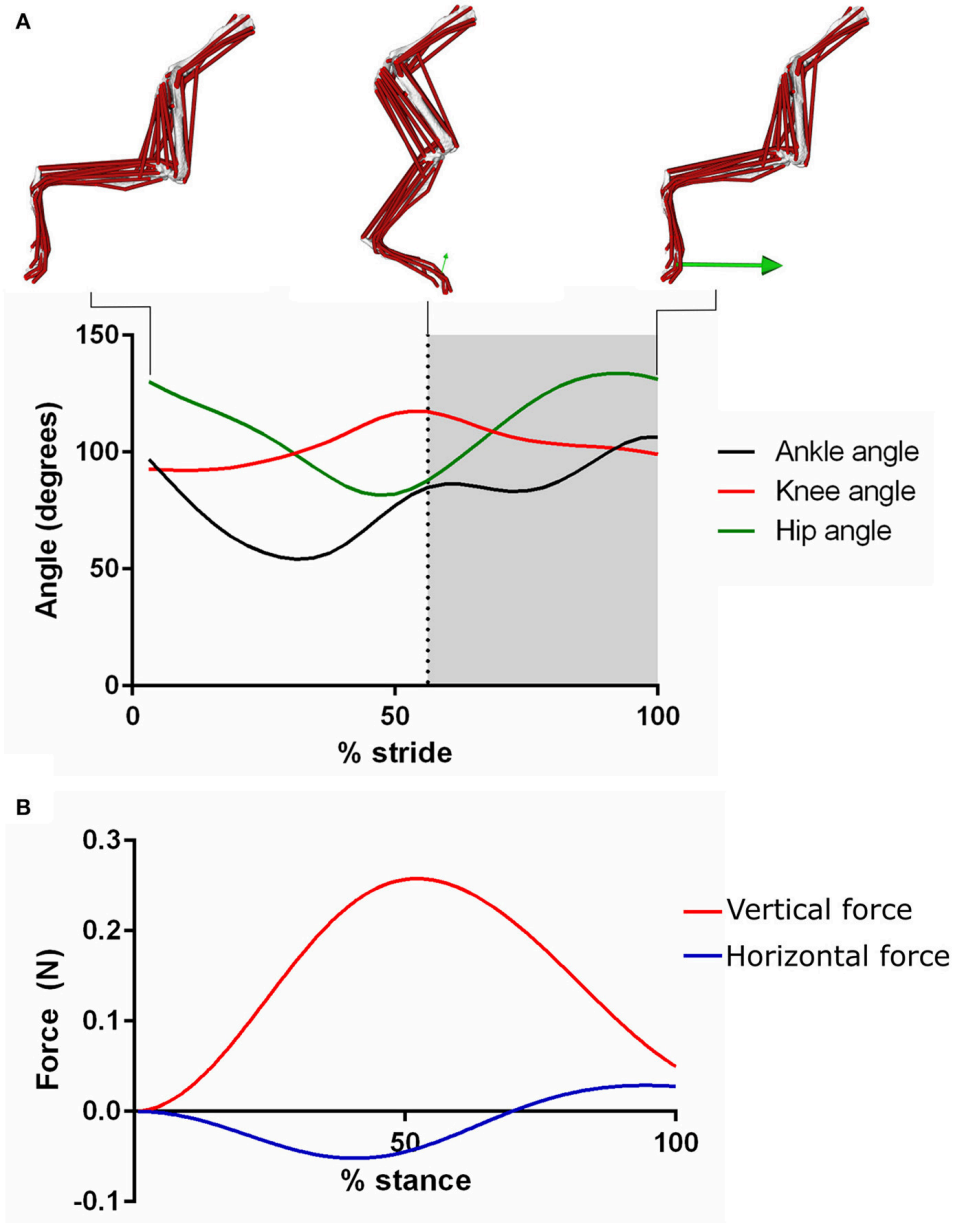
The experimentally derived joint angles **(A)**, and ground reaction forces **(B)** used to create the dynamic simulation of trotting within the mouse hindlimb. The green arrow represents the ground reaction force vector. Positive angles represent hip flexion, knee extension and ankle plantarflexion. Negative angles represent hip extension, knee flexion and ankle dorsiflexion. The gray area indicates the stance phase.

The single stride that fit best within the 95% confidence intervals of both the joint angle and GRF data was used to develop the mouse hindlimb trotting simulation. This stride's duration was 0.128 s, with the transition between the swing and stance phases at 0.072 s (duty factor: 0.44). The experimentally measured joint angles and GRFs for this trial are shown in Figure 5. This mouse was used to scale the musculoskeletal model (see Materials and Methods).

### MTU moment arms

MTU moment arms throughout this simulated stride generally support earlier predictions of mouse hindlimb muscle functions (Charles et al., 2016a), with Semitendinosus (ST), and Rectus femoris (RF) respectively the major hip and knee extensors (in terms of largest moment arms). The results also hint that Pectineus (PECT), with a zero-crossing moment arm (switching between functioning as a hip flexor and extensor) (Figure S1), might provide a stabilizing function during gait.

### Joint moments

**Figure 6:**
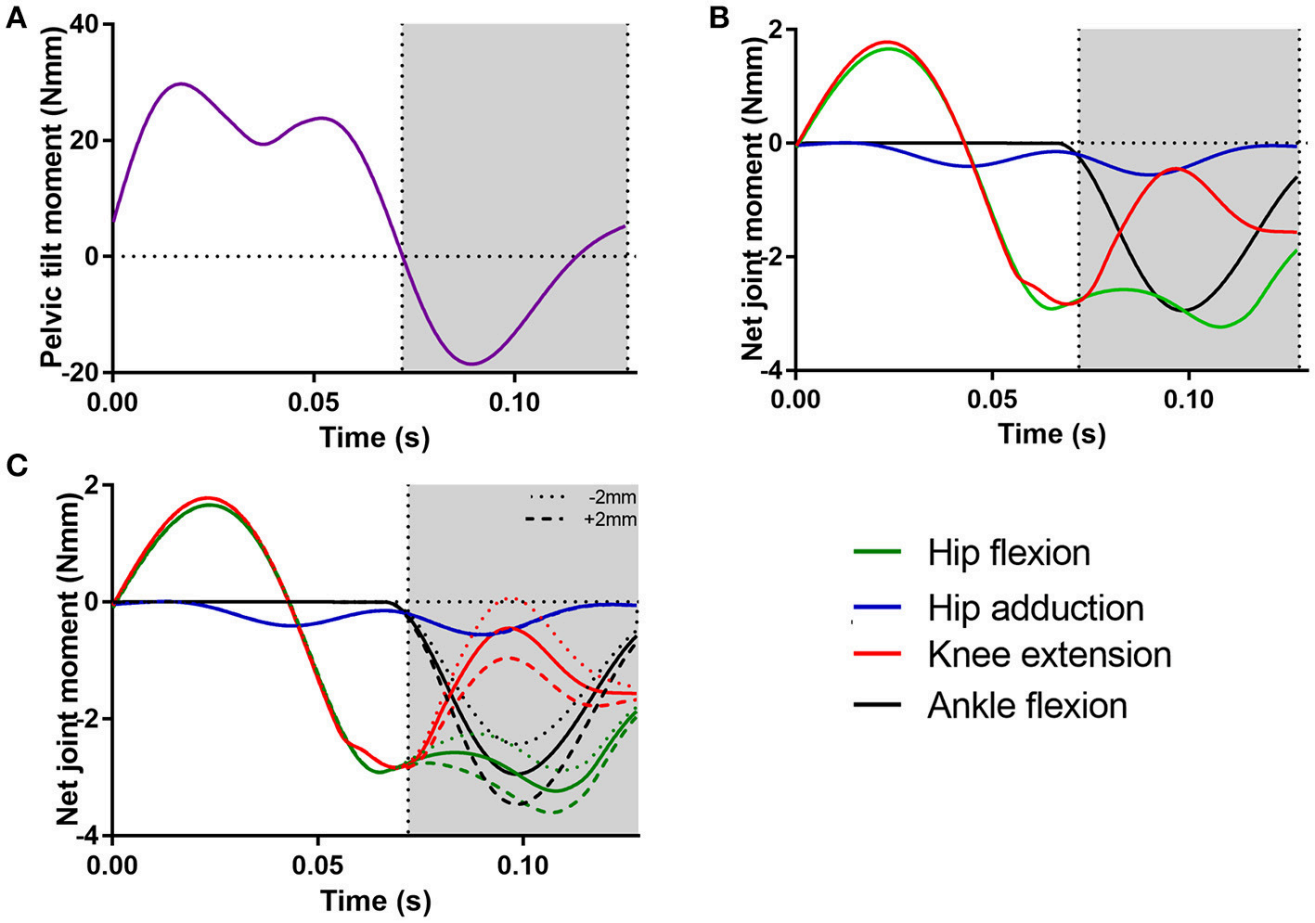
The net joint moments for each unlocked degree of freedom in the model: **(A)** pelvic tilt moment, **(B)** hip flexion, hip adduction, knee extension, and ankle flexion moments. The effect of changing the location of the fixed center of pressure (CoP) of the ground reaction forces (±2 mm along the cranial-caudal axis of the foot) on these joint moments is shown in **(C)**. The gray area indicates the stance phase.

Net moments around the hip, knee and ankle joints, based on the experimentally derived kinematics, were estimated by inverse dynamics (Figure 6). In addition to pelvic tilt (peak moment of 29.70 Nmm; Figure 6A), there were peak hip flexion and knee extension moments during the swing phase (~1.7 Nmm; Figure 6B). There was a peak negative knee extension moment (knee flexion) of −2.80 Nmm just prior to the start of the stance phase. Joint moments were higher during the stance phase, where the hip flexion moment peaked at −3.20 Nmm (hip extension) and ankle flexion peaked at a −2.90 Nmm moment (ankle plantarflexion).

Displacements of the GRF's CoP ± 2 mm along cranial-caudal axis of the pedal segment caused small changes in peak hip flexion, knee extension, and ankle flexion moments around the mid-point of the stance phase (Figure 6C), although the overall patterns of these moments remained similar. CoP movements in the caudal direction (−2 mm) resulted in decreases in peak negative hip, knee and ankle moments (decreases of −0.43, −0.51, and −0.51 Nmm, respectively), whereas movements in the cranial direction (+2 mm) resulted in similar increases in peak negative moments (increases of 0.43, 0.51, and 0.58 Nmm, respectively).

### Muscle activations

**Figure 7:**
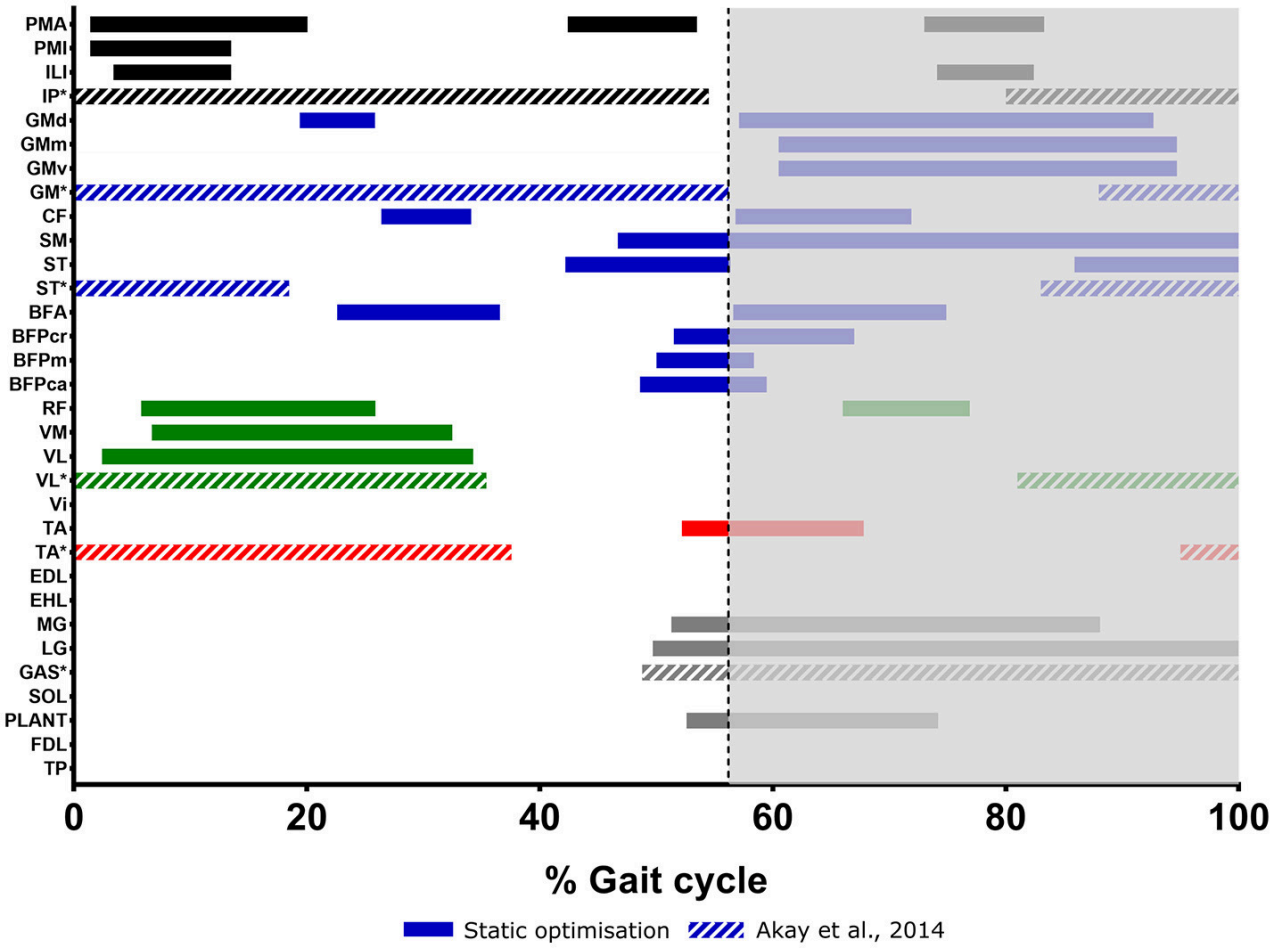
Muscle activations in the mouse hindlimb through one trotting stride, as estimated by static optimization. Measured activations from EMG reported by Akay et al. (2014) during walking gait is shown for comparison (indicated by ^*^IP, Iliopsoas; GAS, Gastrocnemius). The gray area indicates the stance phase.

Figure 7 shows the patterns of activation for the major muscle groups as estimated by static optimization. Most muscles had a single main activation period during the stride, which occurred in either swing or stance phase. The hip flexors and knee extensors were mostly active in the swing phase, although Psoas major (PMA), Iliacus (ILI), and RF showed brief activation periods in the stance phase. Many of the hip extensors were activated late during swing phase to initiate foot strike and continued to be active in early stance phase to extend the hip, and also to flex the knee joint. Tibialis anterior (TA) was the only ankle dorsiflexor estimated to be active during this stride, at late swing/early stance phase. Similarly, the Gastrocnemius muscles (medial and lateral; MG, LG) and Plantaris (PLANT) were the only ankle plantarflexors estimated to be active and were mainly active at late swing phase and through most or all of the stance phase.

### Forward dynamics

The muscle activations from static optimization were used to drive a forward dynamic simulation of a single mouse hindlimb trotting stride, which was used to estimate the mechanical work of individual MTUs. The differences between the experimental hindlimb kinematics and those estimated by forward dynamics are shown in Figure S2A. Many joint angles, other than ankle flexion, matched reasonably well throughout the swing phase, although greater disparities were seen during the stance phase, particularly at the knee and ankle joints (Figure S2B; also see Table S2 for root mean squared errors). Overall, 7 out of 15 quantities in Table S2 showed joint angle errors < 10°. These kinematic differences led to large differences in joint moments, particularly in hip and knee flexion/extension during the swing phase. Differences were smaller in the stance phase (Figure S2C).

### MTU mechanical work

**Figure 8:**
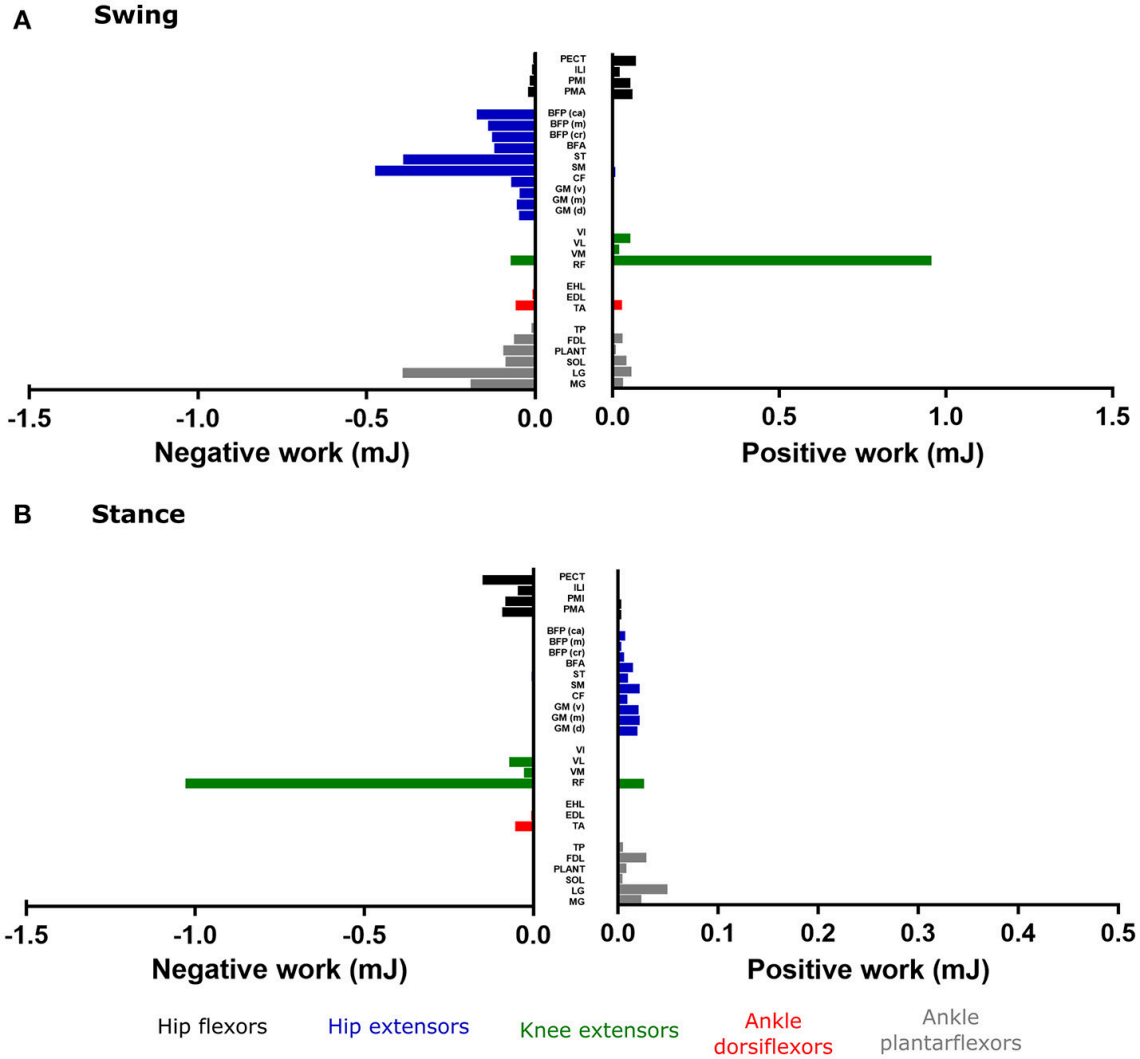
The positive and negative mechanical work generated from each musculotendon unit during the swing phase **(A)** and the stance phase **(B)**, as estimated by static optimization and muscle analysis in OpenSim. Work from hip adductors and ankle everters was negligible, and therefore not shown.

Figure 8 shows the positive and negative MTU work estimated by static optimization and muscle analysis (as opposed to forward dynamics presented above), for the major muscle groups of the hindlimb throughout swing phase (Figure 8A) and stance phase (Figure 8B). The hip extensor and ankle plantarflexor MTUs generated exclusively negative work throughout the swing phase, with Semimembranosus (SM) and lateral gastrocnemius (LG) generating the largest amounts (−0.48 and −0.39 mJ respectively). The RF MTU performed the largest amount of positive work during the swing phase (0.95 mJ), with other MTUs generating relatively little positive work.

Positive MTU work during the stance phase was relatively small during the stance phase, although this was largest in the hip extensor and ankle plantarflexor MTUs. The hip flexor and knee extensor MTUs generated negative work during this part of the stride, with RF generating the largest amount (−1.02 mJ). The ankle everter MTUs produced negligible positive or negative work throughout either swing or stance phase; although it is important to recognize that their eversion moments were prevented by the assumption that the ankle was a simple hinge.

**Figure 9:**
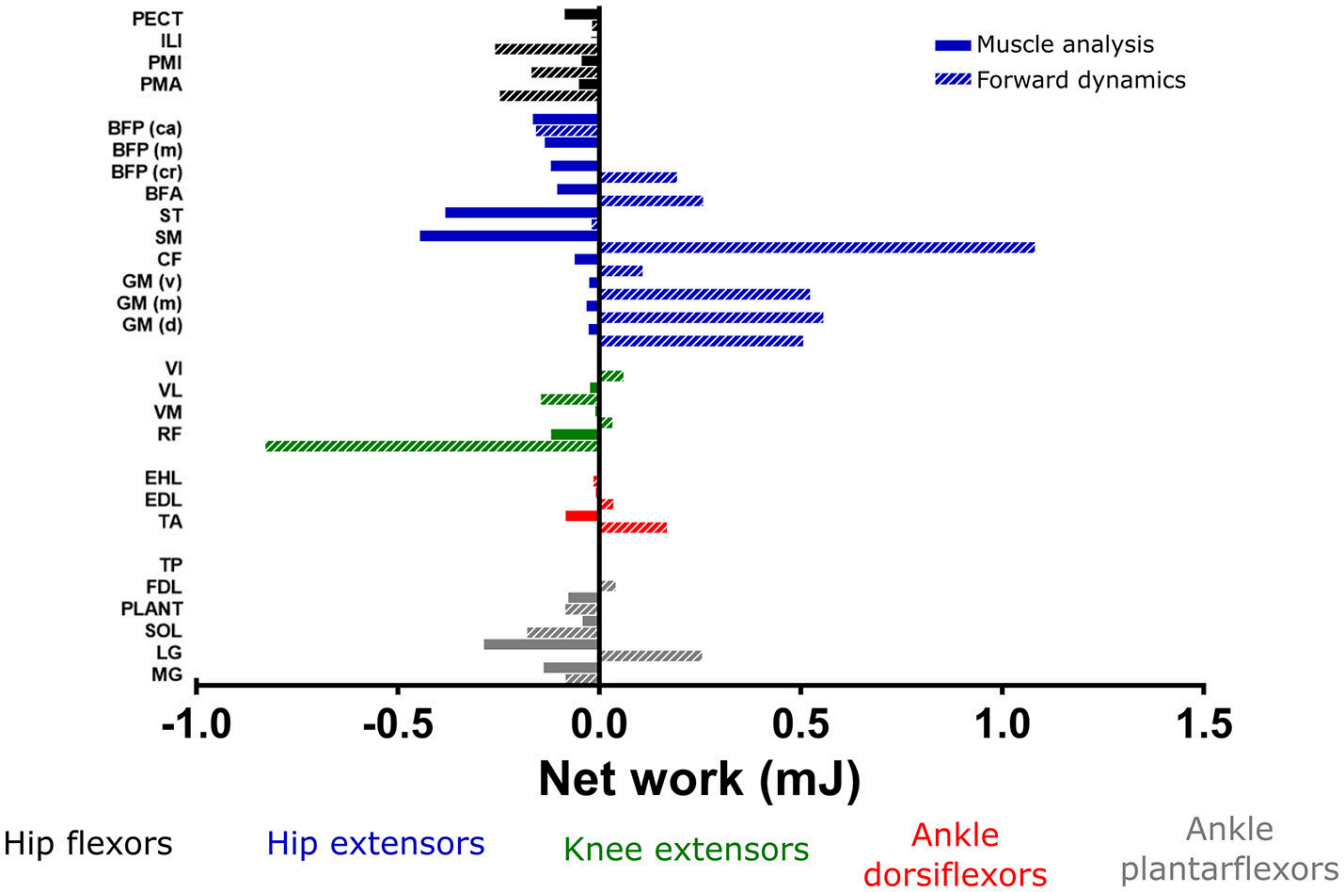
Net mechanical work generated from each musculotendon unit over the whole trotting stride calculated by static optimization and muscle analysis, compared to the values estimated by forward dynamics. See Methods for more details regarding the calculation of mechanical work from these simulations. Work from hip adductors and ankle everters/inverters was negligible, and therefore not shown.

The muscle analysis estimated all MTUs to generate net negative mechanical work across the whole stride (Figure 9), with the negative work in the one part of the stride outweighing the positive work. This is in contrast to the estimates of MTU work from the forward dynamics simulation, which estimated similar patterns of positive/negative work for most muscle groups, except the bi-articular hip extensors/ knee flexors. Here, the hip extensors (especially SM) generated relatively large amounts of net positive work (1.1 mJ) and the knee extensors generated moderate levels of net negative work. The ankle dorsiflexors mostly generated small amounts of net work over the whole stride, however, TA did appear to exert a moderate amount of net positive work (0.2 mJ). The ankle plantarflexors were more variable in their mechanical work output over the whole stride, with LG generating moderate amounts of positive work (0.3 mJ), whereas Soleus (SOL) and MG generated moderate amounts of negative net work (−0.2 and −0.1 mJ respectively).

## Discussion

### Muscle activations

A static optimization analysis was carried out on the musculoskeletal model to estimate the individual muscle moments, and patterns of hindlimb muscle activations during trotting in the mouse hindlimb. The muscle activations (Figure 7) show distinctions between swing and stance phases in terms of the active functional groups, supporting the functional classifications made in our previous research (Charles et al., 2016a,b). It was also possible from this simulation to infer the major MTU from each functional group that was responsible for each major direction of joint rotation.

Of the hip flexor MTUs, PMA, and PMI appeared to contribute equally to hip flexion as they were active at similar periods during swing phase, and produced similar force amounts. However, Pectineus (PECT) was estimated by the simulation to be minimally active during trotting and produced minimal force, suggesting that this MTU may contribute more to long-axis rotational movements at the hip (which were not simulated here) or stability (as per the moment arm analysis in Results above; Figure S1).

All of the hip extensor MTUs were active during trotting, mostly during the late swing and early stance phases, with some continuing to be active during the late stance phase (interestingly, late stance was when their hip extensor moment arms tended to be minimal as per Figure S1; perhaps attributable to increased roles in producing rapid joint excursions rather than large moments). The three portions of Gluteus maximus (GMd, GMm, and GMv) were estimated to be the major hip extensors during the early stance phase (as they produced the most force), whereas SM produced the most force during the late stance phase. As SM is biarticular, crossing the hip and knee joints, it could be that this MTU was functioning to flex the knee at this portion of the stride.

The VL MTU was estimated to be the prime extensor (highest force output- see Figure S3) of the knee joint during the swing phase, with the other knee extensors exerting relatively little force during this part of the stride; however, during the stance phase, RF was the only active knee extensor (corresponding to its large moment arm; Figure S1). The knee extensor MTUs might have been estimated to be more active if any knee adduction/abduction or internal/external rotation were modeled here.

The LG MTU was estimated to be the major ankle plantarflexor during the stance phase of trotting (it exerted the most force), whereas MG produced much less force. The other muscles of this group were less or not at all active, fitting the expectation that together the gastrocnemius MTUs are the main contributors to ankle plantarflexion during dynamic movements, while the other MTUs (such as PLANT and SOL) may be more important during slower movements, or even in providing stability to the ankle.

The ankle dorsiflexors had little estimated activations during most of the swing phase, with only the TA MTU estimated to be active throughout the entire stride. It is almost certain that Extensor digitorum longus (EDL), Extensor hallucis longus (EHL), and Flexor hallucis longus (FHL) would have been more active during trotting if rotations at the pedal joints (i.e., digital flexion/extension) had been included in the model.

Limited detailed data exist in the literature regarding mouse hindlimb muscle activity during locomotion in general, and such information for trotting locomotion is not currently available to our knowledge. Therefore, as a form of preliminary validation for these hindlimb muscle activation patterns estimated by static optimization, we compared select MTU activation periods to those gathered from EMG during walking by Akay et al. (2014) (Figure 7). We found that there were some strong agreements between the two data sets, but also some notable disagreements.

The hip flexors (combined as one Iliopsoas complex) were seen to have one long activation period throughout the whole stance phase in Akay et al. (2014), however our simulation estimated shorter (and in the case of PMA, multiple) activation periods in this part of the stride for the separate hip flexor muscles. Akay et al. (2014) also reported an activation period of the hip flexors in the late stance phase, which overlapped with the estimated activations of PMA and ILI in the same part of the stride. Our model did not estimate any substantial activation periods of the Gluteus MTUs during the swing phase (other than a short activation of GMd), whereas Akay et al. (2014) showed activation of this muscle group throughout the whole swing phase, as well as toward the end of the stance phase, which overlaps slightly with the estimated stance phase activations of our model. The activation of ST during the stance phase was similar between our model and Akay et al. (2014), however, our simulation did not estimate activity at the beginning of the swing phase.

The early swing phase activations of VL were similar between our model and Akay et al. (2014); however, we did not find any activation of this muscle in the stance phase, although another knee extensor, RF, was active around a similar part of the stride. There was no agreement between our estimations and Akay et al. (2014) in terms of the activity of TA across the gait cycle, with our model only estimating an activation period in late swing/early stance phase, but Akay et al. (2014) showing activity in early swing and late stance phase. In contrast, our estimated activation of LG in the stance phase shows an almost perfect match with that of Akay et al. (2014).

Figure 2 shows that the differences between the trotting mouse hindlimb kinematics obtained here, and the walking joint kinematics obtained in Akay et al. (2014) are not drastic, despite some differences in the magnitudes of some joint angles. We therefore judge that the comparison made here between our predicted muscle activations and EMG data from Akay et al. (2014) is sufficient to preliminarily support some of the estimated muscle activations. However, we acknowledge that the estimated patterns of muscle activations presented here represent just one possible set of activations to produce a single trotting stride in the mouse hindlimb, estimated based on just one of many possible optimization criteria (reducing muscle activations squared). The functional redundancy in the mouse hindlimb means that several different activation patterns are able to produce the same functional outcomes.

Furthermore, it is possible that the patterns of MTU activation here may not totally reflect those that would be seen *in vivo*. The lack of many passive structures in this model, such as ligaments (which were at least partially accounted for by reserve actuators), the fact that static optimization does not include the contributions of tendons, the assumption of a fixed CoP of the GRF, as well as the 2D sagittal plane kinematics used to build the simulation, meant that several muscles may have been estimated to be activated differently than they would be *in vivo*. Nevertheless, we contend that this optimized set of muscle activations is informative, and when combined with knowledge of the musculoskeletal architecture and geometry of the mouse hindlimb (Charles et al., 2016a,b), help place previous inferences of MTU functions into a more dynamic and functional context.

### MTU mechanical work

Although musculotendon architecture and geometry, in combination with the estimated muscle activations detailed above, can give an indication of a muscle's function during movement (i.e., the joint rotations it might produce, see Charles et al., 2016a,b), these data are unable to indicate the role of each muscle in distributing the flow of mechanical energy through the limb during a movement (i.e., trotting in this study). Musculotendinous contributions to the flow of mechanical energy throughout swing and stance phase of gait, as well as over the whole stride, can indicate whether an MTU acts as a motor, brake, strut or a spring (Dickinson et al., 2000; Biewener, 2011; Higham et al., 2011; Roberts et al., 2011; Syme and Shadwick, 2011; Rankin et al., 2016). The positive, negative and net mechanical work generated by each MTU actuator, along with the estimated amount of force they produced, were analyzed here to resolve individual MTUs' roles (Figures 8, 9).

The RF MTU was estimated by the simulation to be the primary motor (generated largest amount of positive work) during the swing phase of trotting (Figure 8A), acting to extend the knee. The negative work from the hip extensors and ankle plantarflexors (specifically SM and LG) suggests that these MTUs functioned as brakes during swing phase, possibly to slow down hip flexion and ankle dorsiflexion in preparation for stance.

No MTU was estimated to act as a particularly strong motor during the stance phase, although each uni- and bi-articular hip extensor/ knee flexor MTU generated small amounts of positive work, despite producing high forces (Figures S3B,C). These small amounts of work are correlated with the small knee joint excursions during the stance phase (18°) and the antagonistic action of cranial pelvic tilt with hip extension. This suggests that these MTUs are acting more like struts or stabilizers rather than motors during this part of the trotting stride. Along with the large amount of negative work generated by RF about the knee during stance, the low work but high forces from the hip extensors and knee flexors could be providing a stabilizing function to maintain the crouched limb posture typical of small non-cursorial rodents. It is therefore possible that additional propulsive forces and positive work (motor functions) during trotting in the mouse musculoskeletal system are provided by structures which were unmodeled here, such as MTUs controlling the pelvic tilt DoF (e.g., axial muscles), or the trunk and/or contralateral forelimb.

In other simulation studies (such as Rankin et al. (2016)), distal MTUs (ankle dorsiflexors and plantarflexors) have been concluded to act as mechanical springs, storing energy (negative work), and releasing similar amounts of energy (positive work) later in the stride. Coupled with high amounts of net work generated by more proximal MTU groups, this has led to inferences of a proximo-distal gradient of muscle function, particularly in large quadrupeds and bipeds. This gradient is may be an adaptation for energetically efficient locomotion (Daley et al., 2007). Whether small non-cursorial quadrupeds such as mice benefit from this elastic energy storage in distal muscle tendons and possess anatomical adaptations for energy efficiency has been debated (Pollock and Shadwick, 1994; Bennett and Taylor, 1995; Biewener and Roberts, 2000; Roberts, 2002; Bullimore and Burn, 2005), although by examining muscle architecture data it was postulated that such elastic energy storage could be present in the mouse hindlimb (Charles et al., 2016a). Given the relatively high net negative work of the lateral and medial gastrocnemius MTUs estimated here, the presence of this adaptation is not clearly supported (in the ankle plantarflexors at least) from this whole-MTU analysis. Further studies which decouple muscle fiber and tendon work in mouse hindlimb MTUs could investigate this issue in more detail.

These MTU functional roles were inferred from the static optimization simulation and muscle analysis tool. To test the sensitivity of these estimates, net MTU work amounts over the whole stride were compared to those calculated from a forward dynamic simulation. While some MTUs were estimated to generate similar levels and/or patterns of net work, the most noticeable differences were seen in the hip extensors, which were estimated to generate large amounts of positive work in the forward dynamics simulation, and therefore acted more like motors. Functional inferences for other MTUs did not appreciably change with the different simulations. Any differences in mechanical work output were likely due to the altered kinematics and joint excursions in the forward dynamics compared to the static optimization simulation (possibly due to the lack of tendon compliance in the static optimization solution and the muscle activations input into the forward simulation). However, the inclusion of non-flexor/extensor joint rotations in forward dynamics (which were not obtainable with the current experimental methods) mean that this is could be a more accurate method of calculating mechanical work in future iterations of the model. Alternatively, more sophisticated optimization algorithms incorporating experimental kinematics with optimal control simulations, such as that described by Lee and Umberger (2016), could also be used to estimate MTU work in future studies.

Overall, the patterns of work exhibited by the hindlimb MTUs reported here were qualitatively plausible and mostly consistent with inferences from prior studies (anatomical, neural, biomechanical and physiological) of MTU function in mice and other species (Charles et al., 2016a,b; Rankin et al., 2016). This should stimulate further confidence in and advances of musculoskeletal dynamic simulations more generally in diverse species, as well as mice themselves. Furthermore, the detailed insights into how certain MTUs (notably RF, LG, and the hip extensors/knee flexors) dominate the flow of mechanical energy through the mouse hindlimb could inform studies using mice as preclinical studies of human neuromuscular disorders (but see Hu et al., 2017).

### Residual and reserve actuators

**Figure 10:**
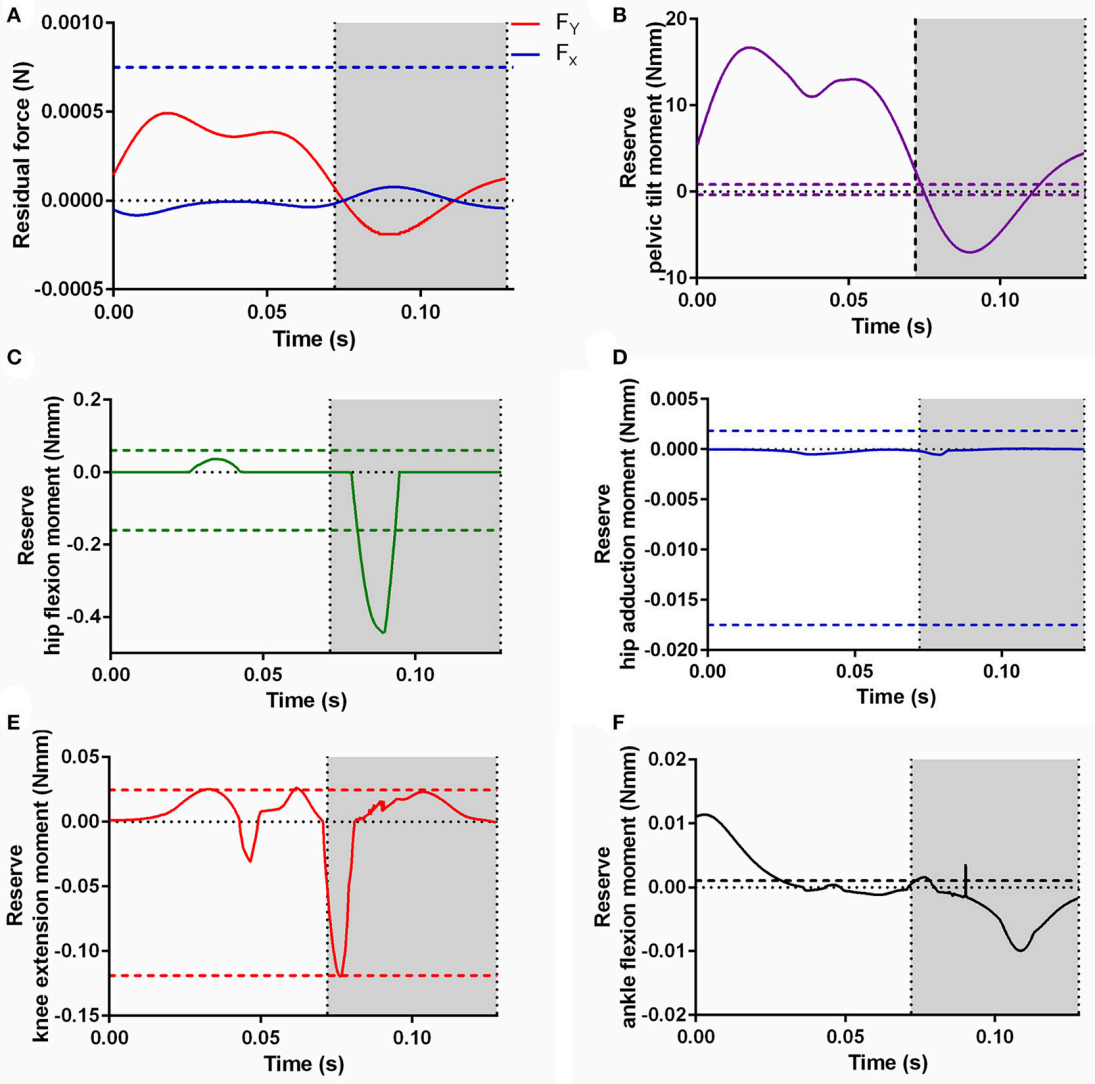
The additional residual actuator forces **(A)** and reserve actuator moments (**B**, Pelvic tilt; **C**, Hip flexion; **D**, Hip adduction; **E**, Knee extension; **F**, Ankle flexion) required to run the static optimization. Horizontal dashed lines represent 5% of the corresponding net joint moment, a reliability threshold for actuator forces/moments recommended by Hicks et al. (2015) for human simulations. Lines representing 5% of F_Y_ **(A)** and ankle plantarflexion moment **(F)** are not shown, as they are too large to fit on the scale. The gray area indicates the stance phase.

To ensure dynamic consistency of the model with experimentally derived GRF and kinematic data during the static optimization and forward dynamic simulations, residual and reserve actuators were appended to the model. Figure 10 shows the forces and moments produced by the residual and reserve actuators appended to the model during the static optimization simulation and their relationship to the net joint moments. The forces produced by residual actuators F_Y_ and F_X_ (accounting for force discrepancies in the vertical and horizontal directions respectively) around the first joint of the model (ground-to-body), did not exceed 5% of the peak GRF in either residual actuator (Figure 10A). These residual forces were necessary due to the lack of a diagonally supportive forelimb or axial muscles/joints, and because body's inertial properties were an approximation.

Although force discrepancies requiring the above residual actuators were expected, they were somewhat low, possibly due to the high forces from the pelvic tilt reserve actuator (Figure 10B). As no muscles were included which controlled this rotation, this reserve actuator accounted for the entire pelvic tilt moment, and likely compensated for the lack of a trunk, a diagonally supportive forelimb or axial muscles/joints in our model (which would help balance pelvic tilt moments) in combination with the residual actuators. The other reserve actuators (hip flexion, hip adduction, knee extension and ankle flexion) were low for most of the stride, although some (hip adduction and ankle flexion) exceeded the recommended limits (Figures 10C–F). In the case of the ankle, the simple modeling of the foot (and CoP) might have contributed to the high reserve actuator moments required here.

**Figure 11:**
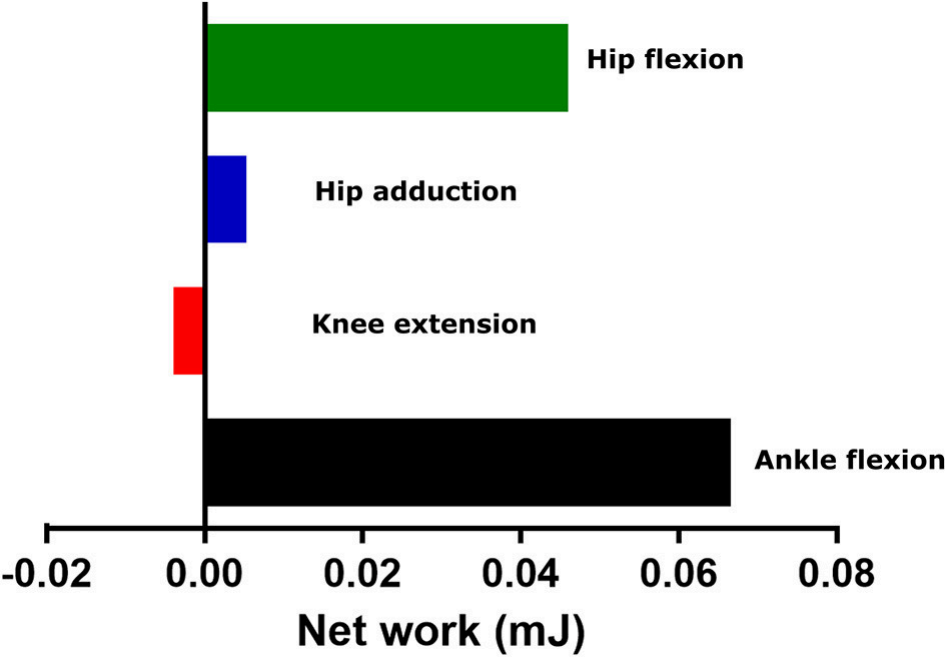
The net mechanical work generated by each reserve actuator and appended to each unlocked degree of freedom in the model over the whole trotting stride. These values give an indication of the compensatory role these actuators were providing to the model across the stride.

Looking at the mechanical work generated by these actuators, as estimated by forward dynamics (Figure 11), may give more insights into what model assumptions/deficiencies these actuators are compensating for. It is possible, and has been discussed elsewhere (Rankin et al., 2016), that reserve actuators compensate for passive structures, such as tendons or ligaments, not included in the model. Passive tissues such as these generally function as struts or springs, generating high forces but little net mechanical work. The reserve actuators here produced relatively small amounts of net mechanical work when compared to the mechanical work of major MTUs acting around those joints as estimated by forward dynamics (Hip flexion- 8% of SM net work; Knee extension- 1% of SM net work; Ankle flexion- 24% of LG net work). This supports the idea that these reserve actuators represent unmodeled passive structures that do not contribute substantially to propulsion or braking during gait. However, the higher proportion of net work by the reserve actuator relative to MTU work in ankle flexion could support our earlier speculations that this actuator was compensating for the simplified modeling of the foot or muscular/anatomical deficiencies around the ankle joint.

While residual actuators can be seen as a “necessary evil” of simulations with incomplete or imprecise experimental data (Millard et al., 2013), we see reserve actuators as a feature of simulations that can give insight onto the non-muscular control of degrees of freedom, rather than a failure of validation. This feature could stimulate future research into how much of a role non-muscular forces and moments have around these joints.

### Limitations of the simulations

Compared to those of the human musculoskeletal system, models and simulations of animal locomotion (bipedal or quadrupedal) are generally less common, with many (such as the one described here) being the first of their kind (Hutchinson et al., 2005, 2015; Johnson et al., 2008; O'Neill et al., 2013; Sellers et al., 2013, 2017; Rankin et al., 2016). Modeling the limbs of animals as small as the mouse, or especially fossil taxa, carries potentially large (and often unknown) amounts of error. While attempts were made here to reduce error, assumptions, and simplifications were made in the construction of this model and simulation, which could impact the results and conclusions drawn here. However, due to the open access nature of this work, there is scope for these limitations and assumptions to be improved upon, which should lead to progressively more valid and reliable models and simulations. The following are the limitations which potentially had the largest effects on the results of the simulations presented here.

#### Center of pressure

In our simulations, the center of ground reaction force application (center of pressure; CoP) was assumed to be located at the mid-point of the distal end of the metatarsal bones, within the pedal (foot) segment, mainly due to technical limitations of the GRF data. This was a reasonable assumption, given the small area of contact between the ground and the phalanges during gait (mice are digitigrade quadrupeds; see Video 1 for trotting example), meaning that the CoP is unlikely to move appreciably during trotting. The use of a fixed CoP was supported with our sensitivity analysis (Figure 6C), which found that the patterns of flexor/extensor moments at the hip, knee and ankle joints were not greatly affected by moving the CoP ± 2 mm (50% of the length of the phalanges) along the cranial-caudal axis of the foot. While peak moments were affected, these findings were consistent with previous, similar sensitivity analyses (Witte et al., 2002; Porro et al., 2017). While this somewhat low sensitivity to CoP location could be unique to small animals with flexed limb postures, these results suggest that potential errors in CoP location do not significantly influence joint moments and have not affected the major findings of this study. Nevertheless, improved GRF and kinematic data could further test this assumption in the future.

#### Lack of trunk and forelimb

Our mouse musculoskeletal model and simulation focused exclusively on MTU mechanics during trotting within the hindlimb and pelvis, ignoring the influences of the contralateral hindlimb, both forelimbs, as well as the torso. While this was, of course, an assumption of the model and not representative of actual mouse anatomy, the lack of these other structures was partly compensated for by the residual actuators included in the simulation (see Methods and Supporting Information). These “hand of god” forces (F_X_ and F_Y_) were applied at the model's center of mass and functioned to account for errors in data collection or the lack of certain anatomical structures. Here, the residual actuators were recruited during the simulation largely to compensate for the lack of trunk and diagonal forelimb.

This assumption could have been at least partially addressed by placing only a fraction of the body weight on the single hindlimb (i.e., reducing the mass of the torso segment of the model). Forelimbs are known to experience greater GRFs and support a greater proportion of body mass than hindlimbs during trotting gaits (Pandy et al., 1988), so determining the extent to which this happens in the mouse during trotting could have more accurately reflected forces within the hindlimb (especially the more proximal joint moments) in this simulation. However, as only hindlimb GRFs were gathered here, it was not possible to compare these to those exerted on the forelimb during the same stride. A more detailed investigation into the distribution of GRFs between the fore- and hindlimbs during fast locomotion in mice, along with the inclusion of axial or forelimb musculoskeletal structures in the musculoskeletal model in future model iterations, would be useful for improving on this assumption. As the representative and other trials were not completely steady-state (e.g., predominantly braking GRFs for the hindlimb in Figures 2D, 5B), this feature also needs improvement in future implementations.

#### Model scaling

The mouse from which the hindlimb kinematic data were measured to create the trotting simulation was not that used to develop the musculoskeletal model (due to the model and the simulation being developed at different times), and there was a slight discrepancy in size and age between these two individuals. These differences were largely accounted for by the scaling tool in OpenSim, although the accuracy may have been hindered by the use of manually determined scaling factors (rather than using motion capture marker placement; a method not able to be used here). The effects of these assumptions were considered to be small or negligible in the mouse hindlimb. Subject-specific models and data would have been an ideal solution, however.

#### Kinematic data

The kinematic data gathered here only measured flexion/extension joint rotations (2D rather than 3D), and so assumed that mouse hindlimb kinematics during trotting locomotion occur exclusively in the sagittal plane (with the exception of estimated hip adduction/abduction angles). In reality, there are likely small degrees of internal/external rotations and even translations and the pelvic, hip, knee, and ankle joints, which if included in this simulation could affect the results and conclusions drawn here. Furthermore, markers were placed on the mice (using white paint) at the approximate joint centers of rotations, which were estimated through palpation. This marker placement introduced unknown inaccuracies into the estimation of the hindlimb joint angles, which were used to create the simulation. More sophisticated methods of gathering mouse hindlimb kinematics (e.g., biplanar fluoroscopy; “XROMM” Brainerd et al., 2010) during locomotion are needed to address these important limitations.

#### The problem of validation

An ideal test of the validity of a musculoskeletal model and simulation would be a direct comparison of the estimated muscle activations to those gathered experimentally using EMG during the same movement of the same individual(s). However, gathering EMG data for trotting mice was out of the scope of this study, and similar data for wild-type mice during overground trotting were not available. Therefore, a comparison to mouse hindlimb muscle activity during treadmill walking (Akay et al., 2014) was made with the muscle activations estimated here (Figure 7). While these onset/offset timings showed broad overlap between the data sets, this comparison is clearly not ideal and there were noticeable disagreements. However, given how the static optimization analysis used here to estimate muscle activations calculated just one set of activations to complete a given task, a complete match to EMG-derived muscle activity was not expected. A similar qualitative comparison was used to validate an ostrich musculoskeletal model and simulation (Rankin et al., 2016), where a reasonable match was found between estimated muscle activations and EMG muscle activity from various birds. However, extensive data are needed to further test the accuracy and reliability of this model and simulation.

Overall, these model limitations are likely to have influenced the results and conclusions presented here. Regardless, given the lack of reference data for mouse functional anatomy, and therefore the lack of any suitable extensive validation, the degree to which these interpretations of MTU functions were affected by these assumptions is difficult to determine. Addressing these limitations in further iterations of the model, i.e., by adding a torso and/or forelimb(s), collecting better kinematic and kinetic data in 3D, or gathering complementary EMG data, is important and will allow us to be more confident in our analyses of the functional anatomy of the mouse musculoskeletal system.

### Future model and simulation potential

As mice are model species for a vast array of research, accurately modeling and simulating their gait has the potential to be of great benefit to a range of scientific fields (e.g., testing how appropriate mice are as “models” for certain human diseases or therapeutic interventions Hu et al., 2017). It will allow for valid, testable calculations of several factors to be made, such as the outcomes of certain therapeutic interventions for human neuromuscular diseases, bone stresses/strains during locomotion, and even the functions of neural sensory feedback loops in maintaining balance and coordination in vertebrates. The model and simulation presented here is a new step in allowing researchers to take a detailed biomechanical modeling approach to these problems.

## Conclusions

Here we created and analyzed static and forward dynamic simulations of mouse locomotion, based on a 44 musculotendon-unit-actuated musculoskeletal model of a mouse's hindlimb and pelvis. Optimization procedures allowed individual muscle activation patterns and levels of mechanical work to be analyzed in detail for a single trotting stride. Calculation of the net mechanical work produced by each individual musculotendon unit during trotting provided further insight into MTU functions within the hindlimb. The apparent dominance of the RF and LG MTUs, as well as the hip extensors, in the flow of mechanical energy through the hindlimb suggest that these muscles should be the main focus of gait studies using mice as preclinical models of human neuromuscular diseases. The high amounts of work by the ankle plantarflexors in particular did not seem to support the presence of a proximo-distal gradient of MTU function within the mouse hindlimb previously inferred from muscle architecture (Charles et al., 2016a); yet simulating different movements (i.e., walking or jumping) as well as creating more detailed simulations and analyses could investigate this further.

Limitations and assumptions made during the creation of the mouse hindlimb model and simulation presented here (and in Charles et al., 2016b) may hinder its accuracy and immediate validity. However, beneficial further developments and validation are made possible by the open access nature and user-extensibility of this work (https://simtk.org/home/mousehindlimb). Therefore, although this work is an important advance for studying mouse hindlimb anatomy and muscle function during locomotion, it only represents the first steps in creating a fully anatomically and dynamically realistic neuromusculoskeletal simulation of the mouse, with potential applications for a wide range of scientific fields.

## Data, code, and materials

The musculoskeletal model used here, and all experimental data, are available from https://simtk.org/home/mousehindlimb.

## Ethics statement

The experiments with mice were approved by the Royal Veterinary College Ethics Committee as study URN 2015 1380 (Biomechanics of mouse gait).

## Author contributions

JC designed the study, carried out the experimental work, conducted all data analysis, and drafted the manuscript. OC carried out the experimental work and helped draft the manuscript. JH conceived of the study, designed the study, coordinated the study, and helped draft the manuscript. All authors gave final approval for publication.

### Conflict of interest statement

The authors declare that the research was conducted in the absence of any commercial or financial relationships that could be construed as a potential conflict of interest. The reviewer TB and handling Editor declared their shared affiliation.

## Supplementary material

Figure S1 Moment arms of the hip flexors **(A)**, hip extensors **(B)**, knee extensors **(C)**, knee flexors **(D)**, ankle dorsiflexors **(E)**, and ankle plantarflexors **(F)** throughout the experimentally derived running stride. The gray area indicates the stance phase.

Figure S2 Comparison between the experimentally derived joint angles (dashed lines) and the joint angles as estimated by the forward dynamics simulation (solid lines) for **(A)** pelvic tilt, hip flexion and hip adduction, and **(B)** knee extension and ankle flexion. Vertical dashed and dotted lines represent the beginning and end of the stance phase of the experimentally derived stride and that estimated by the forward dynamics simulation respectively; i.e., the stride was temporally longer in the simulation. **(C)** Comparison between net joint moments calculated by inverse dynamics, and those from the forward dynamic simulation. The gray area indicates the stance phase.

Figure S3 MTU force output of the hip flexors **(A)**, hip extensors **(B,C)**, knee extensors **(D)**, ankle dorsiflexors **(E)**, and the ankle plantarflexors **(F)** estimated by static optimization and used to drive the forward dynamic simulation. The gray area indicates the stance phase.

Table S1 The properties of the coordinate limit forces appended to each degree of freedom of the mouse hindlimb model, for use in the forward dynamics simulation.

Table S2 Root mean square (RMS) errors (°) of the forward dynamic hindlimb joint angles compared to the experimentally derived kinematics used in the static optimization. Values exceeding 10° are emphasized in bold.

Video S1 High-speed video of trotting mouse used to gather hindlimb kinematics for the representative trial.

Video S2 Static optimization simulation of trotting in the mouse hindlimb. Muscles are colored based on activation; blue, not active; red, active.
