# Supplementary figures and images for: A Dynamic Simulation of Musculoskeletal Function in the Mouse Hindlimb During Trotting Locomotion
Source: Front Bioeng Biotechnol. 2018 May 16;6:61. doi: 10.3389/fbioe.2018.00061 (PMC5964171; doi:10.3389/fbioe.2018.00061)

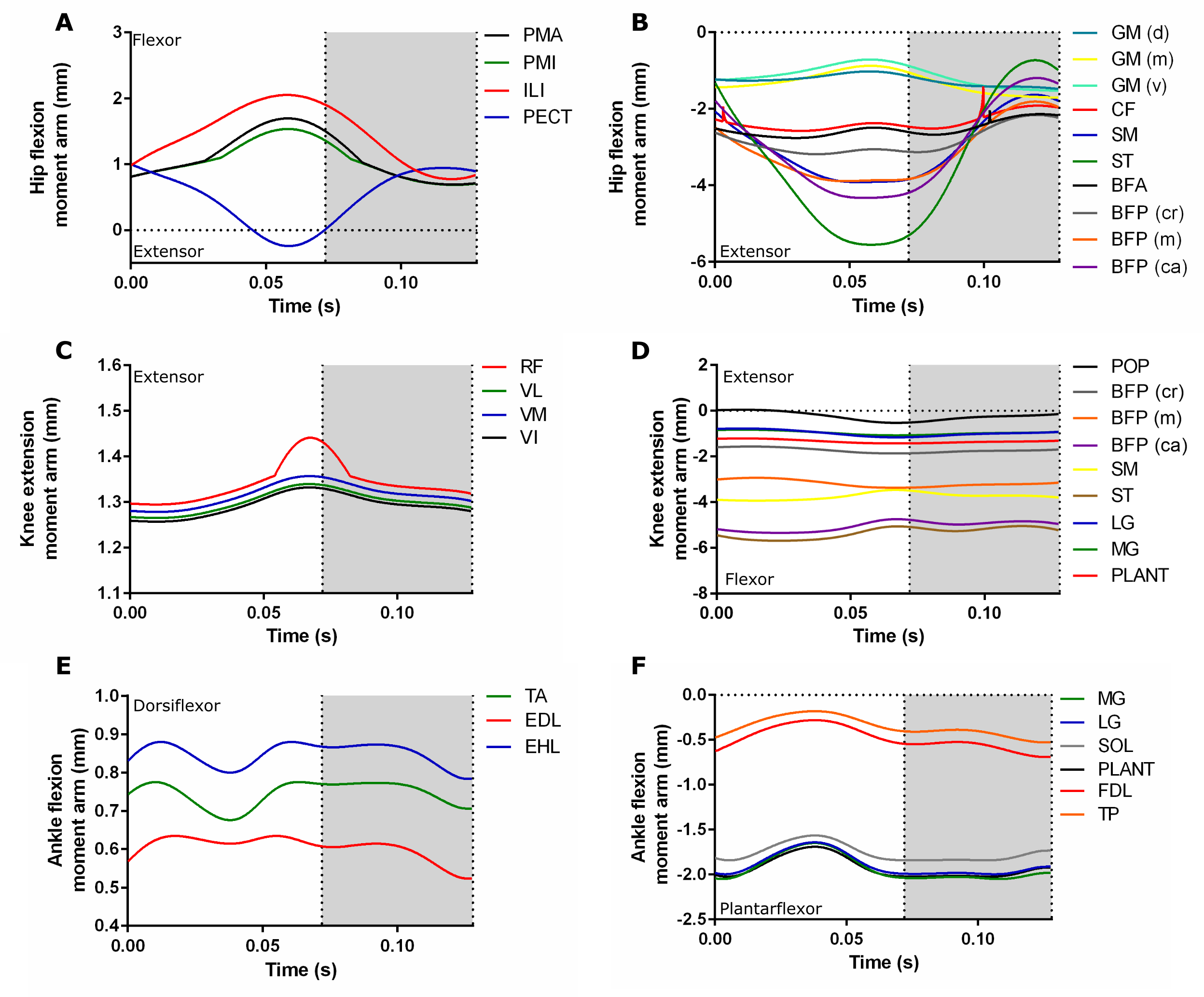

Supplement: Figure S1 — Moment arms of the hip flexors (A), hip extensors (B), knee extensors (C), knee flexors (D), ankle dorsiflexors (E), and ankle plantarflexors (F) throughout the experimentally derived running stride. The gray area indicates the stance phase. [file Image_1.TIF]

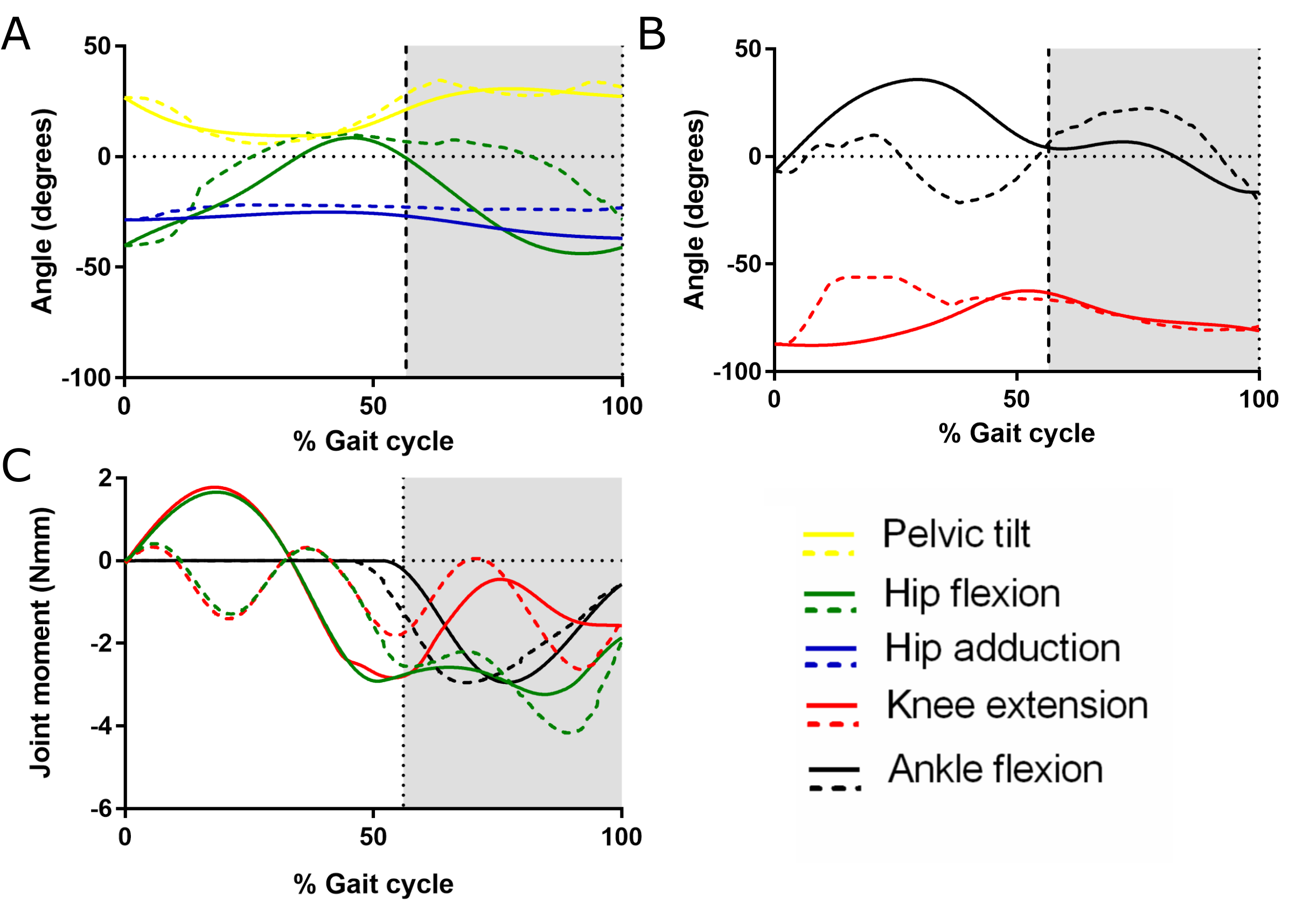

Supplement: Figure S2 — Comparison between the experimentally derived joint angles (dashed lines) and the joint angles as estimated by the forward dynamics simulation (solid lines) for (A) pelvic tilt, hip flexion and hip adduction, and (B) knee extension and ankle flexion. Vertical dashed and dotted lines represent the beginning and end of the stance phase of the experimentally derived stride and that estimated by the forward dynamics simulation respectively; i.e., the stride was temporally longer in the simulation. (C) Comparison between net joint moments calculated by inverse dynamics, and those from the forward dynamic simulation. The gray area indicates the stance phase. [file Image_2.TIF]

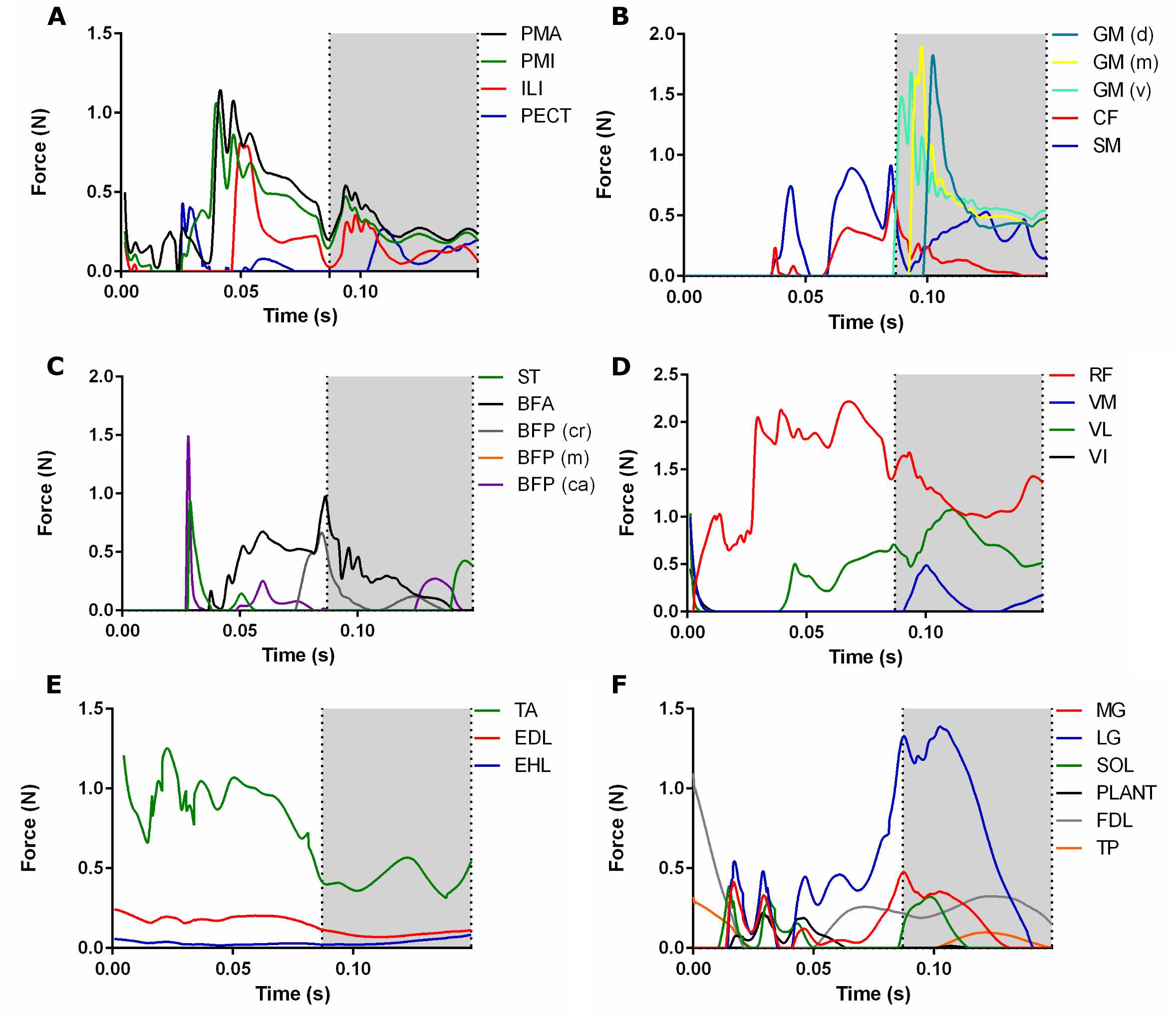

Supplement: Figure S3 — MTU force output of the hip flexors (A), hip extensors (B,C), knee extensors (D), ankle dorsiflexors (E), and the ankle plantarflexors (F) estimated by static optimization and used to drive the forward dynamic simulation. The gray area indicates the stance phase. [file Image_3.TIF]
