# Supplementary material for: A Dynamic Simulation of Musculoskeletal Function in the Mouse Hindlimb During Trotting Locomotion
Source: Front Bioeng Biotechnol. 2018 May 16;6:61. doi: 10.3389/fbioe.2018.00061 (PMC5964171; doi:10.3389/fbioe.2018.00061)
Supplement: Table S1 — The properties of the coordinate limit forces appended to each degree of freedom of the mouse hindlimb model, for use in the forward dynamics simulation. [file Table_1.DOCX]

**Table S1. The properties of the coordinate limit forces appended to each degree of freedom of the mouse hindlimb model, for use in the forward dynamics simulation.**

|  | **Pelvic tilt** | **Hip flexion** | **Hip adduction** | **Knee extension** | **Ankle flexion** |
| --- | --- | --- | --- | --- | --- |
| **Upper limit (°)** | 32 | 9 | -25 | -68 | 36 |
| **Upper stiffness (Nmm/°)** | 100 | 50 | 1 | 0.05 | 0.1 |
| **Lower limit (°)** | 10 | -42 | -40 | -88 | -20 |
| **Lower stiffness (Nmm/°)** | 10 | 1 | 1 | 0.5 | 10 |
| **Damping (Nmm/(°/s))** | 0.1 | 0.001 | 0.1 | 0.001 | 1x10^-5^ |
