# Supplementary material for: A Dynamic Simulation of Musculoskeletal Function in the Mouse Hindlimb During Trotting Locomotion
Source: Front Bioeng Biotechnol. 2018 May 16;6:61. doi: 10.3389/fbioe.2018.00061 (PMC5964171; doi:10.3389/fbioe.2018.00061)
Supplement: Table S2 — Root mean square (RMS) errors (°) of the forward dynamic hindlimb joint angles compared to the experimentally derived kinematics used in the static optimization. Values exceeding 10° are emphasized in bold. [file Table_2.DOCX]

**Table S2. Root mean square (RMS) errors (°) of the forward dynamic hindlimb joint angles compared to the experimentally derived kinematics used in the static optimisation. Values exceeding 10° are emphasized in bold.**

|  | **Pelvic tilt** | **Hip flexion** | **Hip adduction** | **Knee extension** | **Ankle flexion** |
| --- | --- | --- | --- | --- | --- |
| **Swing** | 3.5 | 8.2 | 3.5 | **17.5** | **30.7** |
| **Stance** | 4.4 | **29.7** | 9.6 | 1.7 | **13.8** |
| **Whole stride** | 3.9 | **20.5** | 6.8 | **13.2** | **24.8** |
